# Traditional medicinal plants in South Tyrol (northern Italy, southern Alps): biodiversity and use

**DOI:** 10.1186/s13002-020-00419-8

**Published:** 2020-11-26

**Authors:** Joshua Petelka, Barbara Plagg, Ina Säumel, Stefan Zerbe

**Affiliations:** 1 Faculty of Science and Technology, Free University of Bozen-Bolzano, Bolzano, Italy; 2 Faculty of Education, Free University of Bozen-Bolzano, Bolzano, Italy; 3 Institute of General Medicine, College of Health Care Professions Claudiana, Bozen, Italy; 4 Integrative Research Institute on Transformations of Human-Environment Systems (IRITHESys), Humboldt Universität zu Berlin, Berlin, Germany

**Keywords:** Ecosystem restoration, Human health, Nature conservation, Traditional ecological knowledge, Traditional medicine

## Abstract

**Background:**

Worldwide mountain regions are recognized as hotspots of ethnopharmacologically relevant species diversity. In South Tyrol (Southern Alps, Italy), and due to the region’s high plant diversity and isolated population, a unique traditional botanical knowledge of medicinal plants has flourished, which traces its history back to prehistoric times. However, changes in rural life and culture may threaten this unique biodiversity and cultural heritage. Our study aims to collect and analyze information on native plants used in traditional folk medicine, focusing on the preservation of botanical and cultural diversity.

**Methods:**

Data were collected through a review of published material that documents traditionally used medicinal plants of South Tyrol in order to capture the total diversity of plants and their usage. We evaluated different parameters, comprising the ethnobotanicity index (EI), ethnophytonomic index (EPI), relative frequency of citation (RFC), red list status, and regional legislation with regard to the plant species.

**Results:**

A total of 276 species, including 3 mushrooms and 3 lichens, were identified. These belonged to 72 families, most frequently to the Asteraceae, Rosaceae, and Lamiaceae. The most frequently cited species were *Hypericum perforatum* L., *Urtica dioica* L., and *Plantago lanceolata* L. According to 12 ICPC-2 disease categories, the most frequently treated human health symptoms were from the digestive and respiratory systems as well as the skin. A total of 27 species were listed as endangered, of which 16 are not protected and two are now already extinct. Among the 59 predominantly alpine species, 11 species are restricted to the high altitudes of the Alps and may be threatened by global warming.

**Conclusions:**

Our research revealed that the ethnobotanical richness of South Tyrol is among the highest in Italy and throughout the Alps. Nevertheless, it is evident that biodiversity and traditional knowledge have been heavily eroded. Furthermore, we point out particularly sensitive species that should be reconsidered for stronger protections in legal regulations.

## Introduction

Worldwide mountain regions are recognized as hotspots of ethnobotanical diversity with a high ethnopharmacological importance (e.g., [1–3]). Local species diversity and the historic isolation of mountain settlements lead to a unique ethnopharmacological knowledge that supports health care of local communities [4, 5]. Biodiversity has always been of outmost importance for the provision and new discovery of medical substances [6]. However, an alarming loss of biodiversity is occurring, particularly in mountain regions as they are disproportionably vulnerable to land-use change [7, 8] and climate change [2, 9, 10]. Rising temperatures force mountain plants to move upwards until they reach the highest elevations and become locally extinct (i.e., the ‘summit trap phenomenon’; e.g., [11]).

The current extinction rates of plant species are between 100 and 1000 times greater when compared to natural extinction rates and every 2 years our planet is losing at least one potential major medicinal plant [12]. This rapid rate of extinction and the resulting decline in biodiversity is caused by a combined impact of factors such as urbanization, the overexploitation of natural resources and the pollution of soil, water, and air [13]. Therefore, many international agreements explicitly stress the urgency to document and preserve the floristic and cultural diversity before it is lost (e.g., [14, 15]).

As in other mountain regions in so-called developed countries, changes in the culture and socio-economy of the European Alps in the twentieth century have led to the deterioration of much of the region’s traditional ecological knowledge (see, e.g., [16]) and biodiversity [17, 18]. Despite this process of erosion however, public interest in folk medicine has steadily increased in recent years, highlighting the importance of traditional ecological knowledge for promoting sustainable land management including organic farming, eco-tourism, and eco-gastronomy [19–21]. This can revitalize the relationship between man and nature and help preserving biodiversity and the local cultural heritage in the Alps.

Among the regions of the European Alps, South Tyrol (Southern Alps, Northern Italy) is one of the most interesting from an ethnobotanical standpoint. The interaction of a great floristic richness and a long-lasting cultural history has resulted in a unique ethnopharmacological knowledge within the local population, which can be traced back to prehistoric times [22, 23]. Local literature documents the richness of medicinal plants and related traditional medicinal practices. To our knowledge, no scientific study has jointly addressed both, the ethnobotanical and ecological aspects of medicinal plants, from an interdisciplinary perspective.

Therefore, our study aims to close this gap by (1) compiling and analyzing existing ethnobotanical knowledge on traditional medicinal plants in South Tyrol, (2) assessing the current state of the local diversity of medicinal plants and associated cultural heritage, and (3) highlighting local plant resources of particular interest for regional conservation and/or sustainable agriculture and eco-tourism activities.

## Methods

### Study area

**Fig. 1:**
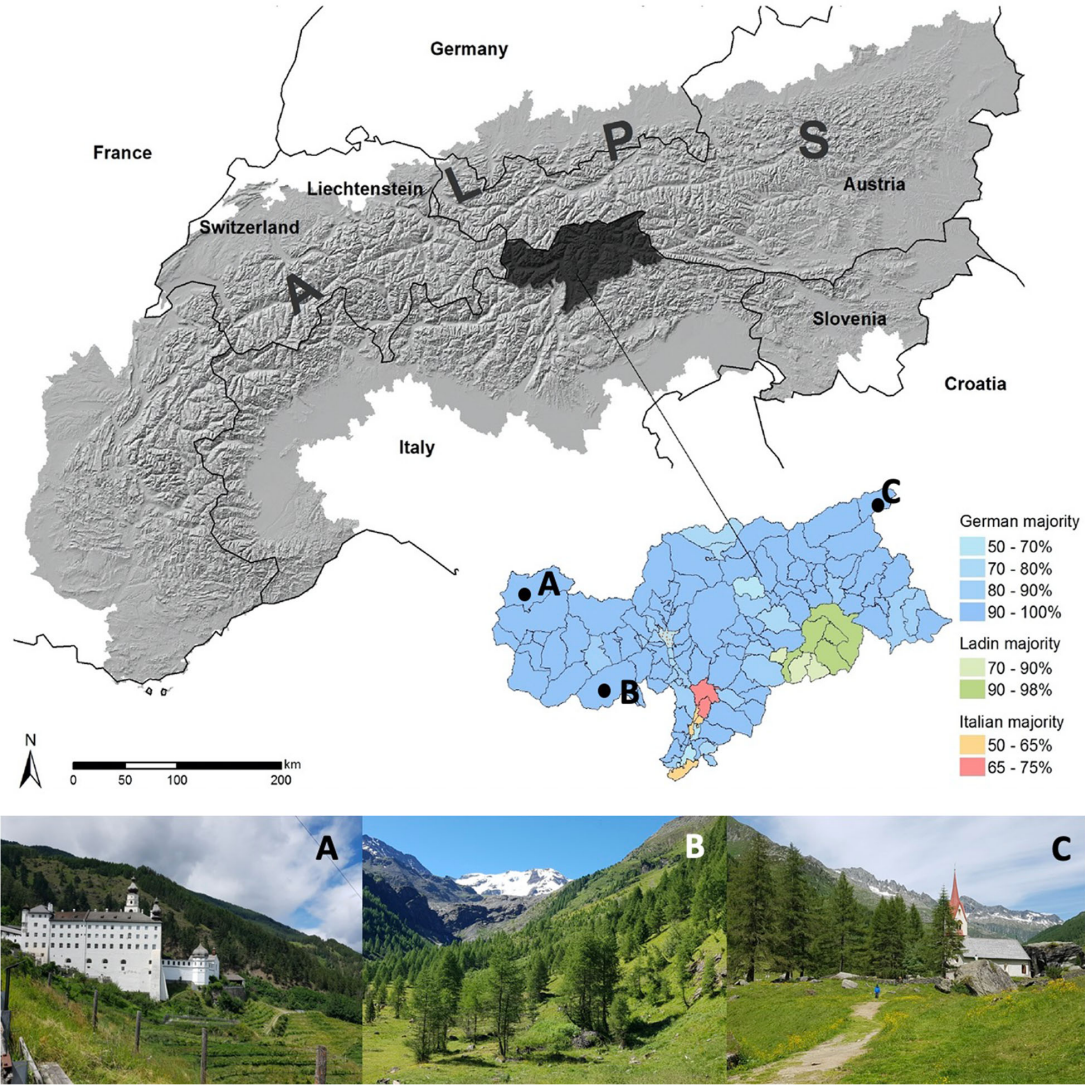
Geographic location of South Tyrol in Northern Italy (grey) [24] and geographical distribution of language groups in South Tyrol according to the census of 2011 [25]. Impressions from South Tyrol landscape: **a** Marienberg Abbey with the highest Benedictine monastery garden in Europe conserve traditional medicinal knowledge. **b** Sulden in the middle of the Stelvio National Park known for the large variety of wild growing medicinal plants. **c** The organic herb farmers of the Ahrntal produce medicinal herbs for regional markets (Photographs: Sylvia Butenschön)

The study area, the Autonomous Province of South Tyrol, is the most northern province of Italy and is situated in the Central Alps, south of the Alps’ main ridge (Fig. 1). The typical mountainous landscape of the region is highly heterogeneous [26] and covers around 7400 km^2^. About 40% of the land is above 2000 m above sea level. Altitudes range from 194 m to a maximum of 3893 m with the Ortler being the highest peak of South Tyrol.

The climate of South Tyrol is intermediate between mountain and Mediterranean climate, with a high relative sunshine duration and a low precipitation compared to the neighboring regions [27]. Over half of the area is forested, predominantly by spruce (*Picea abies*) and pine (*Pinus sylvestris*, *P*. *cembra*), accompanied by mixed forest with beech (*Fagus sylvatica*) and fir (*Abies alba*). One-third of the region is covered by agricultural land and 14% by alpine grassland, glaciers, and rocky areas. Only 3% of the area is classified as settlement area [25]. Typically, the higher altitudes are sparsely populated, while the valleys are characterized by orchards (i.e., apple and grapes) and dense urban areas.

The South Tyrolean Alps harbor 2169 native vascular plant species including many rare and endemic species of which about 25% are endangered and appear in the Red List [28, 29]. Those plant species used for medicinal purposes are very popular in local narratives of the three different linguistic groups that coexist in the South Tyrol; Germans (69%), Italians (26%), and Ladine (5%; see Fig. 1; [25, 30]). Prior to the twentieth century, South Tyrol was characterized by secluded mountain farms and the local population was highly dependent on natural resources for their livelihoods. Native plants were the most important and often the only accessible form of medicine. Thus, over the centuries, unique medical traditions and a variety of vernacular names for medicinal plants developed [31].

In the twentieth century, however, the social, political, and economic structure changed fundamentally and this led to significant ecological degradation and cultural erosion. In the first half of the century, the annexation of the region by Italy and related impacts left deep distortions in the cultural identity of the people [32]. In the 1970s, a rapid economic upswing led to drastic transformations of the social and economic systems in the region that were related to South Tyrol’s political claim of autonomy from Italy. Changes in land use, both the abandonment of mountain pastures and the intensification in the valleys, combined with socioeconomic and environmental processes (tourism, urbanization, pollution, etc.), have dramatically deteriorated the culture and natural environment [22, 32, 33].

### Data collection

Following the PRISMA guidelines [34], we conducted a qualitative review of all publications written in English on alpine medicinal species in the Web of Science by using keywords that cover the medicinal and alpine species (see Appendix A for details). The advanced keyword search (last update March 2020) in the Web of Science revealed 149 references related to medicinal alpine species in the “topic” or “title” fields, more than the 80% (125) have been published since 2008. Numerous papers focus on geographical regions that were not relevant to our study (e.g., India, Nepal, China, USA, Pakistan, Denmark); thus, we filtered the results and kept 31 articles from Alpine European countries such as Italy, Switzerland, Austria, France, Germany, and neighboring alpine regions. In a first step, we screened the titles and abstracts of the remaining articles and eliminated articles that are not related to our topic (10; e.g., genetic surveys or physiological studies of one species). We retained two ethnoveterinarian studies [35, 36] for the next step of the review process. Second, we eliminated any articles that lacked access to a full text version and sent requests to the authors of the most relevant ones. Third, we conducted a full text review of the remaining 21 articles in order to gather relevant information. The whole process was conducted independently by two reviewers who jointly reported a synthesis in Appendix A. Data on medicinal plants of the Alps are mainly based on interviews of local (mostly elderly) people (Appendix A, [22, 23, 26]).

As scientific literature in Web of Science mostly did not cover our study region, we reviewed other published and unpublished material through local library research and through recommendations from local medicinal herb experts. In so doing, we documented traditionally used medicinal plants of South Tyrol in order to capture the total diversity of plants and usages in our study area. Almost all literature was published between 1988 and 2018, including books for the non-expert public and other documents that focused exclusively on the medical usage of plants of local relevance. Thus, the literature covers the period of the upcoming herbal medicine trend in the 1990s and is based mainly on reviving knowledge from old folk medicinal practices (Appendix A). The widespread use of popular books that sometimes lack a certain scientific rigor, however, but reflects the knowledge base of local communities that in other studies was addressed by semi-structured interviews (Appendix A; [31]). This knowledge was passed on from parents to children, often from mothers to daughters. The current increasing interest in medicinal species today, however, cannot rely on the exchange between the generations and often relies on popular science books and websites about medicinal herbs and medicinal plants. Physicians and pharmacists who are interested in herbal remedies often use recognized textbooks as important sources of information about herbs [37]. Altogether, 17 sources were used, including 16 books and one unpublished work (see Appendix A). The 17 sources were comprised of three types: (1) cultural literature (*n* = 7), (2) prescription books (*n* = 9), and (3) scientific manuscripts (*n* = 2; [31], Appendix A, 42). Furthermore, the level of originality of literature was classified into three categories: (1) original information based on interviews or authors’ expert knowledge (*n* = 7), (2) secondary literature that compiled information from the local literature (*n* = 8), and (3) a mix of both (*n* = 3; see details in Appendix A). Only references on South Tyrol were included in further analysis (Appendix C).

Plant species given in the references and their nomenclature where vetted through the online platform “Flora Fauna Südtirol” [38] for vascular plants, the “Information System on Italian Lichens” [39], and the Italian Mycological Association [40]. Finally, the nomenclature was standardized according to “The Plant List” [41]. Data acquired for each plant species included vernacular names, plant parts used, use categories, medicinal use, temporal changes in use, the pharmacological or phytochemical evidence on medicinal use, cultivation, natural habitat, as well as endangerment and protection status. Vernacular names are important in the ethnobotanical cosmos as medicinal plants are known, collected and traded as products with local names in folk taxonomies (e.g., [42, 43]).

### Data analysis

We assessed the total diversity and various uses of medicinal plants by reviewing their general usage and the procurement of species, medicinal use of species, as well as pharmacological or phytochemical evidence for the medicinal healing effect of species. We recorded a number of citations per species (Fig. 3). These parameters were highlighted from both the ecological and cultural perspective. Species diversity comprises the number of used species, botanical and fungal families, growth forms, and altitudinal habitat range. We recorded usage and procurement of species, the (plant) parts most frequently used, whether the plant is collected in the wild or cultivated, as well as whether the plant is used often or has been abandoned. The general use possibilities were grouped into (1) medicinal use, (2) alimentary, (3) veterinary, (4) spiritual, (5) cosmetic, and (6) domestic (Table 1).

**Table 1:** Use versatility of traditional medicinal plants of South Tyrol

Use type	Count	Most frequent plant families	Examples of plant species
Medicine	276	Asteraceae, Rosaceae	*Arnica montana*, *Hypericum perforatum*, *Equisetum arvense*, *Peucedanum ostruthium*, *Plantago lanceolata*, *Sambucus nigra*
Alimentary	128	Rosaceae	*Gentiana lutea*, *Juniperus communis*, *Pinus cembra*, *Rosa canina*, *Sambucus nigra*, *Thymus pulegioides*
Veterinary	70	Asteraceae	*Cetraria islandica*, *Dryopteris filix-mas*, *Geranium robertianum*, *Larix decidua*, *Leontopodium nivale*, *Peucedanum ostruthium*
Spiritual	55	Asteraceae	*Arnica montana*, *Crataegus monogyna*, *Peucedanum ostruthium*, *Hypericum perforatum*, *Juniperus communis*, *Salix alba*, *Sambucus nigra*
Cosmetics	44	Asteraceae	*Betula pendula*, *Leontopodium nivale*, *Matricaria chamomilla*, *Pinus mugo*, *Urtica dioica*, *Trifolium repens*
Domestic	43	Fagaceae, Pinaceae and Rubiaceae	*Betula pendula*, *Equisetum arvense*, *Fraxinus excelsior*, *Galium aparine*, *Larix decidua*, *Malva sylvestris*, *Pinguicula alpina*

We compared the variation of the number of citations, vernacular names, use versatility, and number of medicinal uses between wild and cultivated species; between species with different protection status (i.e., non, partially protected and protected); between species of different Red List categories (e.g., extinct, extinct in the wild, critically endangered, endangered, vulnerable, nearly threatened, least concern); and between different growth forms (i.e., herbs or woody species) and pharmacological or phytochemical evidence of medicinal effects (i.e., positive or negative effect and not investigated). We used the Kruskal-Wallis *H* test followed by the Dunn post-hoc test. We also conducted statistical analyses with the open-source software package R, version 3.3.2 (R Foundation for Statistical Computing, Vienna, Austria).

We sorted and categorized medicinal plant use and ailments treated according to the International Classification of Primary Care (ICPC-2) [44]. By doing so, we differentiated between 12 disease categories (Table 3). Based on [45], the ICPC categories are more suitable for ethnopharmacological studies than other classifications. Pharmacological or phytochemical evidence of the medicinal healing effect of plant substances and medicinal products was determined by monographs from the German Commission E [46] and of the European Medicines Agency (EMA; [47]). These categories are also in line with many ethnobotanical studies, as previously reported by [48].

**Table 2:** Endangered and protected medicinal plants species used in traditional folk medicine in the region of South Tyrol according to [29]

Red-list status	Count	Species
Extinct	2	*Eryngium amethystinum*, *Eryngium campestre*
Critically endangered	1	*Cetraria islandica*, *Mentha pulegium*, *Usnea barbata*
Endangered	4	*Cyanus segetum*, *Dipsacus fullonum*, *Marrubium vulgare*, *Rosa montana*, *Usnea dasypoga*
Vulnerable	8	*Adiantum capillus-veneris*^a^, *Allium ursinum*, *Gentiana lutea*^b^, *Hyoscyamus niger*, *Ilex aquifolium*^a^, *Anacamptis morio*^a^, *Primula matthioli*^a^, *Quercus robur*
Near threatened	11	*Althaea officinalis*, *Centaurium erythraea*^*a*^, *Drosera rotundifolia*^*a*^, *Galega officinalis*, *Leonurus cardiaca*, *Lilium bulbiferum*^*a*^, *Malva alcea*, *Menyanthes trifoliata*, *Nasturtium officinale*, *Nepeta cataria*, *Salix pentandra*
Least concern	238	*Abies alba*^b^, *Aquilegia einseleana*^a^, *Arnica montana*^b^, *Botrychium lunaria*^a^, *Cyclamen purpurascens*^a^, *Gentiana acaulis*^a^, *Gentiana punctata*^a^, *Lilium martagon*^a^, *Lycopodium clavatum*^b^, *Primula auricula*^a^, *Primula elatior*^a^, *Primula glutinosa*^a^, *Primula veris*^a^, *Primula vulgaris*^a^, *Anemone vernalis*^a^, *Ruscus aculeatus*^b^
Data deficient	2	*Alchemilla alpina*, *Alchemilla xanthochlora*
Not evaluated	11	*Aesculus hippocastanum*, *Fomitopsis officinalis*, *Lamium galeobdolon*, *Heracleum sphondylium*, *Lepidium sativum*, *Fomitopsis betulina*, *Ribes petraeum*

^a^ = protected, and ^b^ = partially protected. See complete list in Appendix **C**

For our quantitative analysis, we calculated the ethnobotanicity index (EI: percentage of useful plants from the total flora of the region; see [49] and the ethnophytonomic index (EPI: ratio between reported plants with vernacular names and the total flora of the studied region; see [50]). The latter indicates the richness of people’s knowledge of local plants.

## Results

### Qualitative and quantitative characterization of medicinal species

We named a maximum of 155, a minimum of 18, and an average of 72 plant species per source. On average, the prescription books (mean 72) mentioned more plant species than the books on regional history (mean 60). Four of the 17 references are dated to before 2000, while 13 were published after 2000. Thus, the number of books on the topic after 2000 has tripled. This increase reflects the new social trend of herbal remedy use [51].

We identified a total of 275 native medicinal species that are used (Appendix C), including 204 herbs, 28 trees, 28 shrubs, 9 ferns, 3 mushrooms, and 3 lichens. The species belonged to 72 families, of which Asteraceae, with 32 species, was most frequent, followed by Rosaceae (25), Lamiaceae (18), Apiaceae (15), and Brassicaceae (10). For mushrooms, it was Fomitopsidaceae and for lichens Parmeliaceae. About 21% (59 species) of all species are classified as biogeographically Alpine while the remaining species have a cosmopolitan character. The species most frequently cited in our literature sources were *Hypericum perforatum* and *Urtica dioica* (cited in more than 90% of used sources) followed by *Plantago lanceolata* (cited in more than 85% of used sources; Fig. 3). Furter, 211 plants were collected in the wild, while 64 plants were cultivated in traditional home gardens or on agricultural land. The plant parts most frequently mentioned in literature for their potential use were leaves (41%), flowers, and buds (28%) as well as bulbs and roots (17%). Less commonly used were fruits (7%), bark and resin (5%), and seeds (2%).

At least 17 plant species were reported to be no longer used in South Tyrol (i.e., *Artemisia vulgaris*, *Euphrasia officinalis*, *Galium verum*, *Pimpinella saxifraga*, *Adiantum capillus*-*veneris*, *Botrychium lunaria*, *Sedum roseum*, *Fomitopsis betulina*, *Polygala chamaebuxus*, *Viola biflora*, *Mutellina adonidifolia*, *Primula matthioli*, *Primula auricula*, *Antennaria dioica*, *Biscutella laevigata*, *Beckwithia glacialis*, *Silene vulgaris*). In terms of health benefits, among these species, there are only two species (*Antennaria dioica*, *Euphrasia officinalis*) with negative effects and one species (*Mutellina adonidifolia*) with a positive effect that has been proven by pharmacological or phytochemical evidence. There are no studies available for 80% of the species that are no longer used. All of the species are wild and 76% are herbs. Four species (*Adiantum capillus*-*veneris*, *Botrychium lunaria*, *Primula auricula*, and *Primula matthioli*) are protected species; *Adiantum capillus-veneris*, *Primula matthioli*, and the mushroom *Fomitopsis betulina* are classified as vulnerable or nearly threatened species at the Red List (Table 2).

We found that use versatility was high among the recorded species (see Appendix C). In addition to medicinal applications, 46% of the species were also used in alimentary settings, 25% for veterinary purposes, 20% for spirituality and cults, 16% as cosmetics, and 16% for domestic purposes (Table 1). The species with the highest versatility (i.e., 5 out of the 6 above-mentioned purposes) were the perennial herbs *Alchemilla xanthochlora*, *Artemisia absinthium*, *Equisetum arvense*, *Hypericum perforatum*, *Lilium bulbiferum*, *Urtica dioica*, and *Valeriana officinalis*; the dwarf shrub *Thymus pulegioides*; and the woody species *Betula pendula*, *Corylus avellana*, *Quercus petraea*, *Q*. *pubescens*, *Q*. *robur*, and *Sambucus nigra* (Fig. 3). About one-third of the species (34%) were reported for their exclusive use in only one of the categories while another third (31%) were used for two purposes, 19% for three, and 10% for four different purposes. The so-called *Heublumen* (“hay flowers”) mix is a blend of flowers, seeds, smaller leaves, and stem pieces of various mowed grassland plants.

Plants were most frequent used for the digestive system, skin, and for the respiratory system. These medicinal applications also included the highest number of useful plants (Table 3). Other relevant remedies included general and non-specific disorders (11%), the musculoskeletal system (10%), the urinary tract (8%), as well as the neurological and circulatory system (both 8%). Fewer plant species were used to treat disorders of pregnancy and childbirth, or for family planning (5%). Even fewer were used to treat disorders of the eye (3%), the endocrine system, the nutritional system and metabolism (3%), or for psychological issues (2%). A very high percentage of plants (70%) were used as multi-contextual remedies for several disorders (2–5 categories). The remaining species (14%) were useful in more than five categories and thus were often referred to as universal remedies (in German: “Allheilmittel” or “Heil aller Schäden”). Among these, we found *Equisetum arvense*, *Juniperus communis*, and *Peucedanum ostruthium* (Fig. 3).

**Table 3:** Medicinal use spectrum of traditional medicinal plants in South Tyrol, classified into 12 human disorder categories based on the International Classification of Primary Care (ICPC-2) [44]. The category nutraceuticals is based on plants that have been reported as either being eaten or consumed as a beverage for their medicinal use

Disorders	Plants	Most frequent plant families	Examples of plant species	Nutraceutical food plants
Digestive	152	Asteraceae	*Achillea millefolium*, *Centaurium erythraea*, *Gentiana lutea*, *Peucedanum ostruthium*	91
Skin	133	Asteraceae	*Chelidonium majus*, *Equisetum arvense*, *Hypericum perforatum*, *Plantago spp*.	65
Respiratory	128	Asteraceae	*Cetraria islandica*, *Primula glutinosa*, *Pimpinella saxifraga*, *Pinus mugo*	73
General and unspecified	102	Asteraceae	*Arnica montana*, *Pinus cembra*, *Pimpinella major*, *Thymus pulegioides*	63
Musculoskeletal	93	Asteraceae	*Arnica montana*, *Larix decidua*, *Potentilla anserina*, *Stachys sylvatica*, *Symphytum officinale*	43
Urology	80	Asteraceae	*Achillea moschata*, *Alchemilla alpina*, *Juniperus communis*	45
Neurological	76	Asteraceae	*Crataegus monogyna*, *Humulus lupulus*, *Hypericum perforatum*	34
Cardiovascular	74	Rosaceae	*Achillea millefolium*, *Allium spp*., *Crataegus monogyna*, *Leonurus cardiaca*, *Viscum album*	51
Pregnancy, Childbirth, Family Planning	49	Asteraceae	*Adiantum capillus-veneris*, *Artemisia vulgaris*, *Geranium robertianum*	33
Eye	29	Orobanchaceae	*Daucus carota*, *Euphrasia spp*., *Pimpinella saxifraga*,	14
Endocrine/Metabolic and Nutritional	26	Rosaceae	*Cichorium intybus*, *Taraxacum campylodes*	19
Psychological	19	Equisetaceae and Rosaceae	*Melissa officinalis*, *Pinus cembra*, *Valeriana officinalis*, *Sedum roseum*	17

For 42% of the recorded species (*N* = 111), we found evidence of their medicinal significance in the German database [46]. For 79 of these species, the medicinal purpose has been validated, while for 33 it was rejected. This means that these 33 species are not curative or have severe and even toxic side effects when used. In the European database [47], the medicinal significance of 57 species (21%) were assessed, 215 (78%) were not listed, and 3 species (1%) were registered that their medicinal significance was under discussion.

Species with a higher number of citations (cf) and vernacular names (vn) in folk medicinal literature, as well as more use versatility (uv), or a greater number of medicinal uses (mu) have been subjected to scientific studies that have proven positive effects more often (Kruskal-Wallis Chi^2^_cf_ = 63.5, *p*_cf_ < 0.0001; Chi^2^_vn_ = 25.1, *p*_vn_ ≤ 20090.0001; Chi^2^_uv_ = 22.9, *p*_uv_ < 0.0001; Chi^2^_mu_ = 33.2, *p*_mu_ < 0.0001; see Fig. 4). However, the majority (59%) of native South Tyrolean medicinal species remains understudied.

**Fig. 2:**
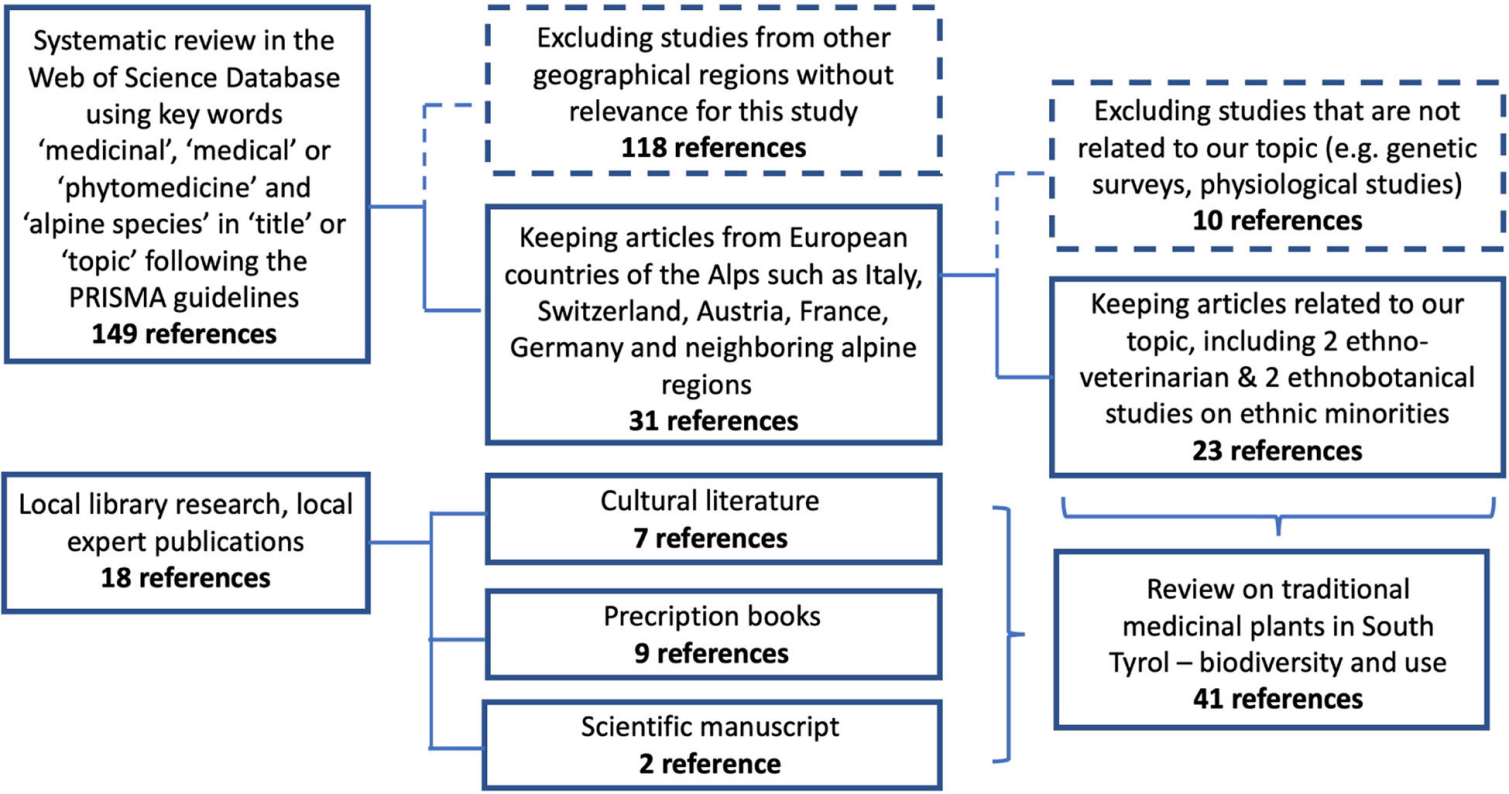
Systematic literature review using PRISMA guidelines (for details see Appendix A)

**Fig. 3:**
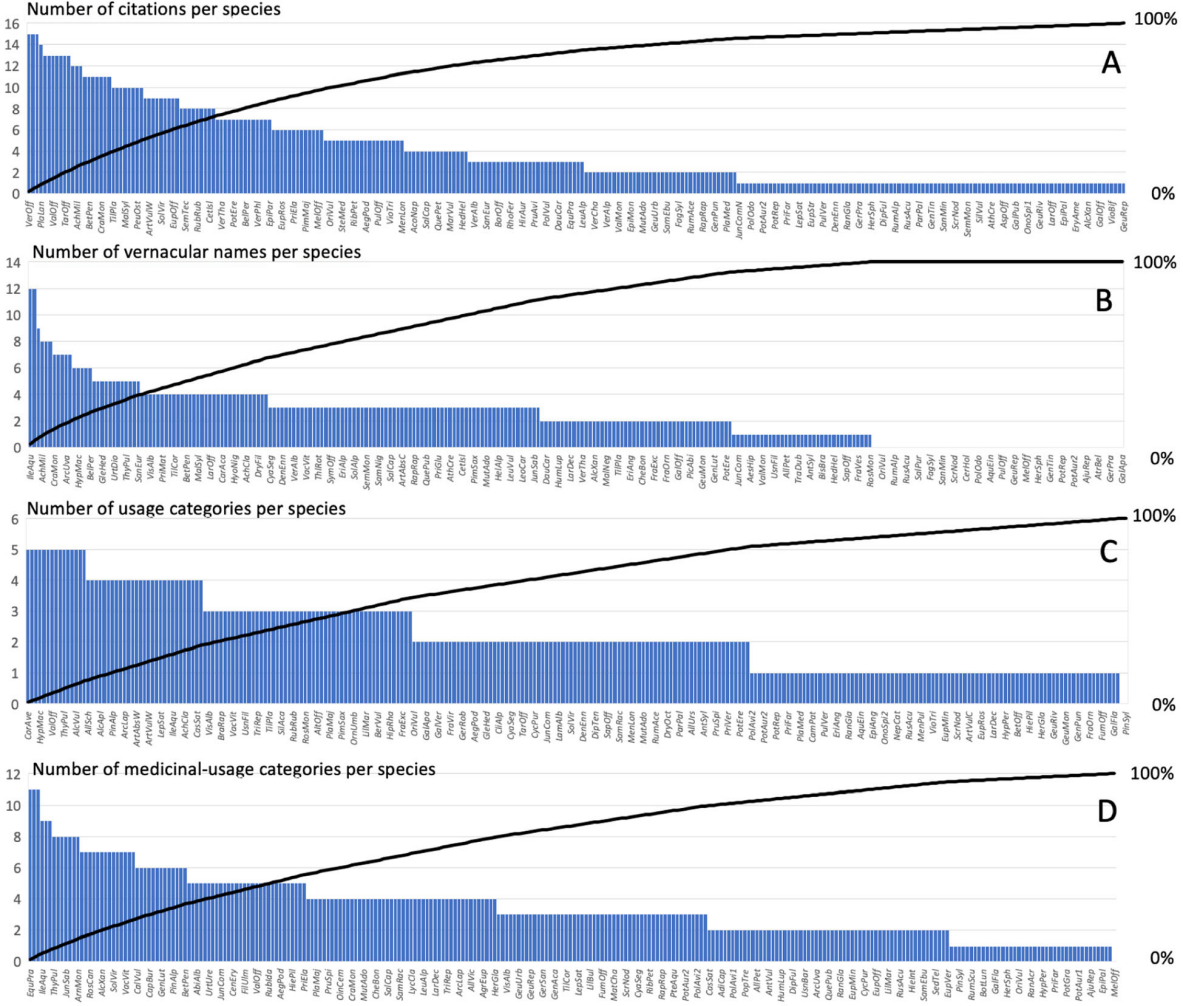
The most frequent cited traditional medicinal plant species in South Tyrol. The number of citations per species (**a**), vernacular names (**b**), usage categories (**c**), and medicinal-usage categories (**d**) are given. The abbreviation of species names consists of the first three letters of the genus name and the first three letters of the specific name (e.g., *HypPer* = *Hypericum perforatum*; *AlcVul* = *Alchemilla vulgaris*). The Pareto line is also given. Full list is provided in Appendix C

We found a total of 714 vernacular names used in traditional South Tyrolean folk medicine that referred to 276 actual species (Appendix B). Thirty-seven of them were quoted with one vernacular name and a few species even had more than 10 names—for example, 17 names for *Achillea millefolium*, *Hypericum perforatum*, and *Juniperus communis* and 18 names were found for *Alchemilla xanthochlora* (e.g., “Frauenhilf,” women’s help, herb to treat gynecological disorders) and *Arnica montana* (e.g. “Donnerblüml,” thunder flower, protection from thunderstorms or “Wundkräutl,” wound herb, herb for wound treatment) (Fig. 3 and Appendix B, C). For 66 species, no local names were indicated.

Frequency of citation in folk medicinal literature (cf), number of vernacular names (vn), or number of medicinal uses (mu) are not related to the growth form of the species (Kruskal-Wallis Chi^2^_cf_ = 7.6, *p*_cf_ = 0.270; Chi^2^_vn_ = 4.4, *p*_vn_ = 0.622; Chi^2^_mu_ = 9.5, *p*_mu_ = 0.149). Woody species have a significant higher number of use versatility (uv; Chi^2^_uv_ = 29.1, *p*_uv_ < 0.001***; see Fig. 5).

### Ethnobotanicity and ethnophytonomic index

Two thousand one hundred sixty-nine vascular species are native to South Tyrol [28]. Considering the large number of vernacular names for the medicinal plants in the local flora, the calculated ethnophytonomic index (EPI) (0.102) indicates that popular knowledge about native plants was very rich. The ethnobotanicity index (EI) for medicinal plants was 12%, thus about every eighth plant of the local flora is used in folk medicine.

### Assessment of vulnerability

According to the regional Red List [29], a total of 24 species are listed as endangered, including one that is critically endangered (*Mentha pulegium*), four endangered (*Cyanus segetum*, *Dipsacus fullonum*, *Marrubium vulgare*, and *Rosa montana*), 9 vulnerable, and 11 nearly threatened species (Table 2). Two species (*Eryngium amethystinum* and *Eryngium campestre*) were listed as extinct. With regard to the protection status, we found that about 95 of the medicinal species (35%) were under the protection status of the regional legislation (Table 2). Of those, 19 species (7%) were strictly protected, while five (*Abies alba*, *Arnica montana*, *Gentiana lutea*, *Lycopodium clavatum*, and *Ruscus aculeatus*) were under partial protection, meaning that permission for extraction from nature or use can be granted through exceptions issued by the regional authority.

**Table 4:** Medicinal species in South Tyrol that are predominately alpine according to [52] and with the altitudinal range alp = alpine, sniv = sub-nival, suba = sub-alpine, mont = montane, and coll = colline; in bold = predominantly alpine occurrence (according to [22])

Altitudinal range	Count	Plant species
alp-sniv	5	*Achillea atrata*, ***Achillea moschata***, *Geum reptans*, *Beckwithia glacialis*, *Veronica alpina*
Alp	2	*Dryas octopetala*, *Silene acaulis*
suba-sniv	3	*Leucanthemopsis alpina*, ***Hieracium intybaceum***, *Salix serpyllifolia*
suba-alp	14	***Aconitum napellus***, ***Alchemilla alpina***, *Allium victorialis*, ***Artemisia mutellina***, ***Cetraria islandica***, *Erigeron alpinus*, ***Geum montanum***, *Juniperus communis* var. *saxatilis*, *Mutellina adonidifolia*, ***Primula glutinosa***, ***Anemone vernalis***, ***Rhododendron ferrugineum***, *Sempervivum montana*, *Thlaspi rotundifolium*
mont-alp	20	*Achillea clavennae*, ***Arctostaphylos uva-ursi***, ***Arnica montana***, *Clinopodium alpinum*, *Gentiana acaulis*, ***Gentiana lutea***, *Gentiana punctata*, ***Leontopodium nivale***, ***Peucedanum ostruthium***, ***Pinus cembra*** *Potentilla aurea*, *Primula auricula*, *Rumex alpinus*, *Sedum atratum*, *Sempervivum tectorum*, *Soldanella alpina*, ***Vaccinum myrtillus***, ***Vaccinium vitis-idaea***, *Viola biflora*
mont-suba	6	*Botrychium lunaria*, *Chenopodium bonus-henricus*, *Erica carnea*, ***Pinus mugo***, *Rosa pendulina*, ***Veratrum album***
coll-alp	5	*Antennaria dioica*, *Biscutella laevigata*, *Globularia cordifolia*, *Parnassia palustris*, *Primula farinosa*
coll-suba	4	***Calluna vulgaris***, ***Carlina acualis***, *Polygala chamaebuxus*, ***Thymus pulegioides***

Frequency of citation in folk medicinal literature (cf), use versatility (uv), or number of medicinal uses (mu) are not related to the protection status of the species (Kruskal-Wallis Chi^2^_cf_ = 2.8, *p*_cf_ = 0.224; Chi^2^_vn_ = 2.2, *p*_vn_=0.331; Chi^2^_mu_ = 3.7, *p*_mu_ = 0.158). Non-protected species have significantly more vernacular names (vn; Chi^2^_uv_ = 7.4, *p*_uv_ = 0.024*; see Fig. 5e). Frequency of citation in folk medicinal literature (cf), number of vernacular names (vn), use versatility (uv), or number of medicinal uses (mu) are not related to the status of the species on the local Red List (Kruskal-Wallis Chi^2^_cf_ = 6.6, *p*_cf_ = 0.471; Chi^2^_vn_ = 8.9, *p*_vn_ = 0.263; Chi^2^_uv_ = 8.2, *p*_uv_ = 0.316; Chi^2^_mu_ = 13.2, *p*_mu_ = 0.067).

Out of the 276 medicinal plants, about 59 species (21% of all native medicinal species) can be phytogeographically considered to be alpine (Table 4). However, some of those (e.g., *Arnica montana* and *Vaccinium vitis-idaea*) can also be found at lower elevations. In our study, we identified at least ten medicinal species that are restricted to the upper alpine zone (> 2600 m a.s.l.) which are the perennial herbs *Achillea atrata*, *Achillea moschata*, *Dryas octopetala*, *Geum reptans*, *Hieracium intybaceum*, *Leucanthemopsis alpina*, *Beckwithia glacialis*, *Silene acaulis*, and *Veronica alpina* and the woody species *Salix serpyllifolia*.

## Discussion

### South Tyrol as a hotspot of traditional medicinal plants

Our study explores the local pharmacopoeia of South Tyrol, a region which has thus far been poorly investigated in ethnopharmacological studies when compared to neighboring regions [53–55].

Herbs dominate South Tyrolian medicinal species, followed by woody species. Ferns, mushrooms, and lichens are less frequently used (Appendix C). Woody species have a higher overall use versatility when compared to herbs (Fig. 5f) which is related to the variety of plant parts used such as leaves, bark, roots, or flowers.

Similar to other studies on traditional medicinal plant use (e.g., Appendix A [22, 23]), the South Tyrolian species are predominately cosmopolitan, only 20% are alpine (Table 4). Biogeographically, 16 of the 20 most cited plants are also found in other parts of Europe, while four grew only in alpine areas. Thirty-five percent of the medicinal species are classified as threatened or protected species (Table 2). In general, non-protected species are of higher importance for medicinal or veterinarian use than protected species (Fig. 5e). Cultivated medicinal species are dominant and thus, in contrast to other more pristine mountain regions (e.g., [56] in Himalaya or [57] in Ethiopian Highlands), overexploitation is not a current issue in South Tyrol. This has also been reported for other regions with long phytomedicinal traditions (e.g., [58] from Central China or [59] from the Balkan Mountains). However, extinction processes due to overharvesting may already have run their course in the Alps. Medicinal species that are currently in use mainly grow in the bottom of valleys rather than at high alpine zones [22].

**Fig. 4:**
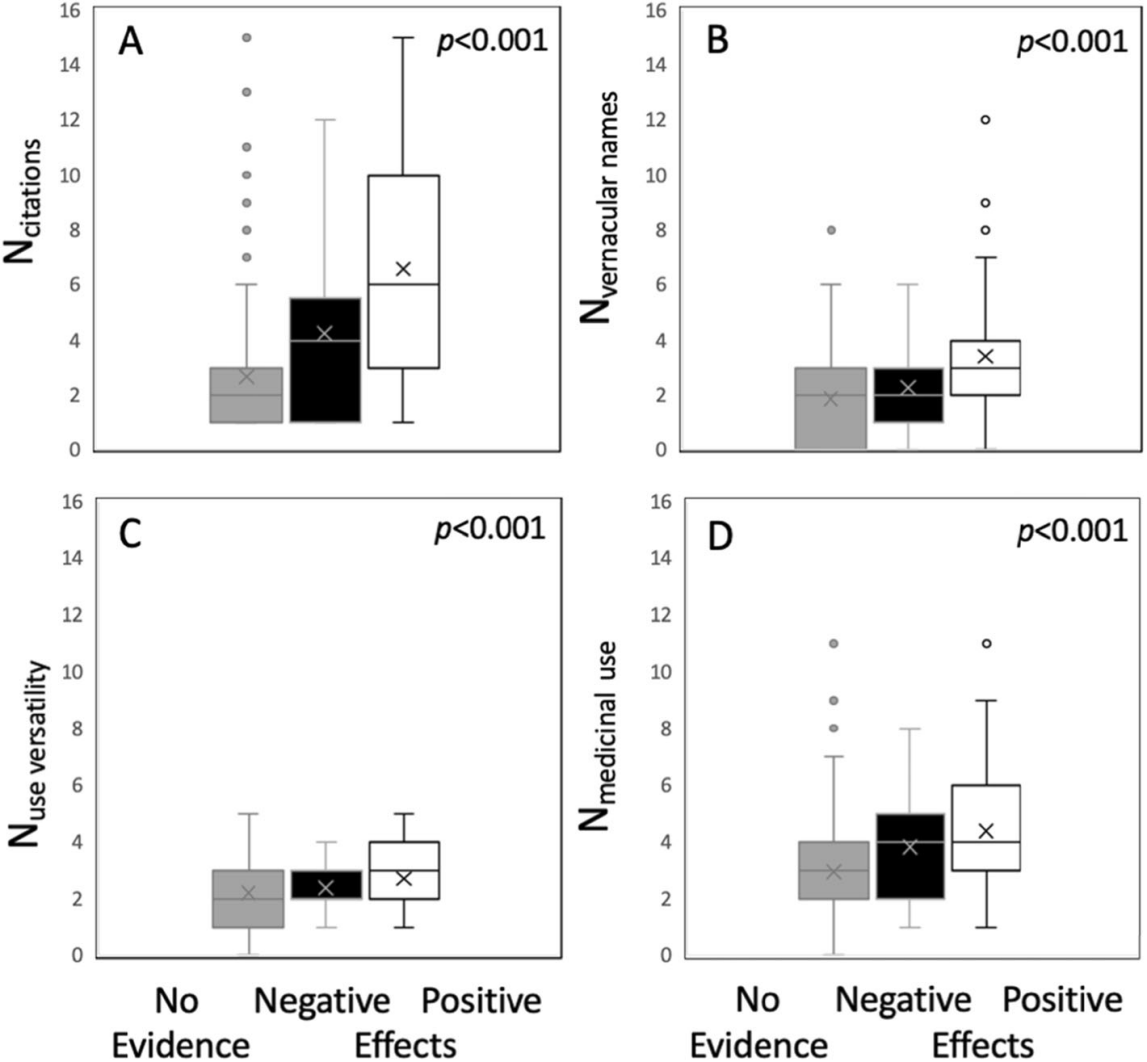
Comparison of species without pharmacological or phytochemical evidence, with evidence of negative and of positive effects regarding number of citations in folk medicinal literature (**a**), number of vernacular names (**b**), overall use versatility (**c**), and medicinal use versatility (**d**) in South Tyrol. For results of the Kruskal-Wallis rank sum test, see text

Twelve percent of all South Tyrolean species are used in traditional folk medicine. This proportion ranks among the highest in the European alpine regions and is similar to the Basque country and the neighboring Swiss region of Prättigau. The proportion is twice as high as in the Albanian Alps (Table 5). The ethnobotanicy index for South Tyrol is higher than in other mountain regions of Italy and demonstrates a broad local knowledge of medicinal species. Thus, despite land use changes and industrialization processes, local ethnopharmacological heritage is still being preserved among elders and inhabitants of rural areas (e.g., [31]) and in popular science books (Appendix A). South Tyrol’s EI is similar to the global average value for medicinal plants (12%), see [64].

**Table 5:** Overview of various ethnobotanical studies of mountain regions in Europe

Reference	Country/Region	Study area	Number of species		EI	Source
			Total flora	MP		
[60]	Central- southern Italy (Molise)	378 km^2^	ca. 800	70	6.0 %	54 Informants
[61]	Eastern Italian Alps (Friuli- Venezia Giulia)	5,700 km^2^	ca. 3335	177	5.3 %	n.d. Informants
[53]	Western Italian Alps (Liguria)	86 km^2^	ca. 1500	105	7.0 %	65 Informants
[54]	Northern Italian Alps (Lombardy)	896 km^2^	ca. 2185	184	8.4 %	328 Informants
Present study	Northern Italian Alps (South Tyrol)	7,400 km^2^	ca. 2169	275	12.0 %	17 Literature sources (a.o. 81 informants in [31])
[48]	North-west Spain (Basque Country)	802 km^2^	ca. 1133	139	12.3 %	207 Informants
[62]	Albanian Alps (Kosovo)	3,500 km^2^	ca. 1609	98	6.1 %	91 Informants
[63]	Eastern Switzerland (Prättigau)	610 km^2^	ca. 1414	204	14.4 %	91 Informants

*MP* medicinal plants, *EI* ethnobotanicity index

A high number of vernacular names for medicinal species underlines that popular knowledge of medicinal plants in South Tyrol is still well consolidated (Fig. 3; Appendix B). However, the generally lower IE values in some regions (Table 5) may indicate a more rapid process of cultural erosion and a loss of ethnopharmacological knowledge [60, 65]. Ethnobotanical studies on wild and cultivated plants used as food and medicine by the other ethnic minority in the Alps reported that traditional knowledge on plant names and uses was limited to the older generations (Appendix, [22, 23]).

When estimated by the rarely used ethnophytonomic index (EPI 0.10), popular knowledge of wild species is widespread, exceeds the previously reported value for Sondrio (0.06; [3]), and is comparable to the Central Alps (0.10; [54]).

By comparing the 20 most cited plants with those that had the most vernacular names, we can observe two general trends. Firstly, 10 of the most cited plants were not among the top 20 plants with the most vernacular names but they have become popular nowadays, e.g., *Matricaria chamomilla*, *Plantago lanceolata*, *Rosa canina*, and *Urtica dioica*. The high popularity of these species in the region may be attributed to the fact that they appear in most phytotherapy books. Secondly, the 20 plants with the most vernacular names include a higher number of alpine plants (7), which is typical for the alpine environment of the study area, e.g., *Carlina acaulis*, *Leontopodium nivale*, and *Peucedanum ostruthium*. The count of local names is more likely to reflect the original medicinal plants, whereas the most cited plants probably also represent modern plants adapted by knowledge transfer, i.e., neighbors, books, and seminars. Thus, the list of plants with the most vernacular names may be the better scale for evaluating the traditional importance of a plant (Fig. 3 and Appendix B and C).

The high number of vernacular names per species (Appendix B) also illustrates the linguistic diversity of our study area, which is represented by the three official languages: German, Italian, and Ladin and manifold local dialects [66]. Overall, the findings from both indices reinforce the assumption that is highlighted in the ethnobotanical survey from [22], that traditional ethnobotanical knowledge is prevalent and well documented in the popular literature while the number of traditionally used species in the area is high.

Established herbal pharmacopoeias conserve local knowledge on medicinal species and function as profound repositories for buried knowledge that is currently assisting the revitalization of natural medicine. As a concequence, a variety of drugs that are derived from plants that were known to ancient civilizations and used throughout the millennia are today being included in modern pharmacotherapy [67].

**Fig. 5:**
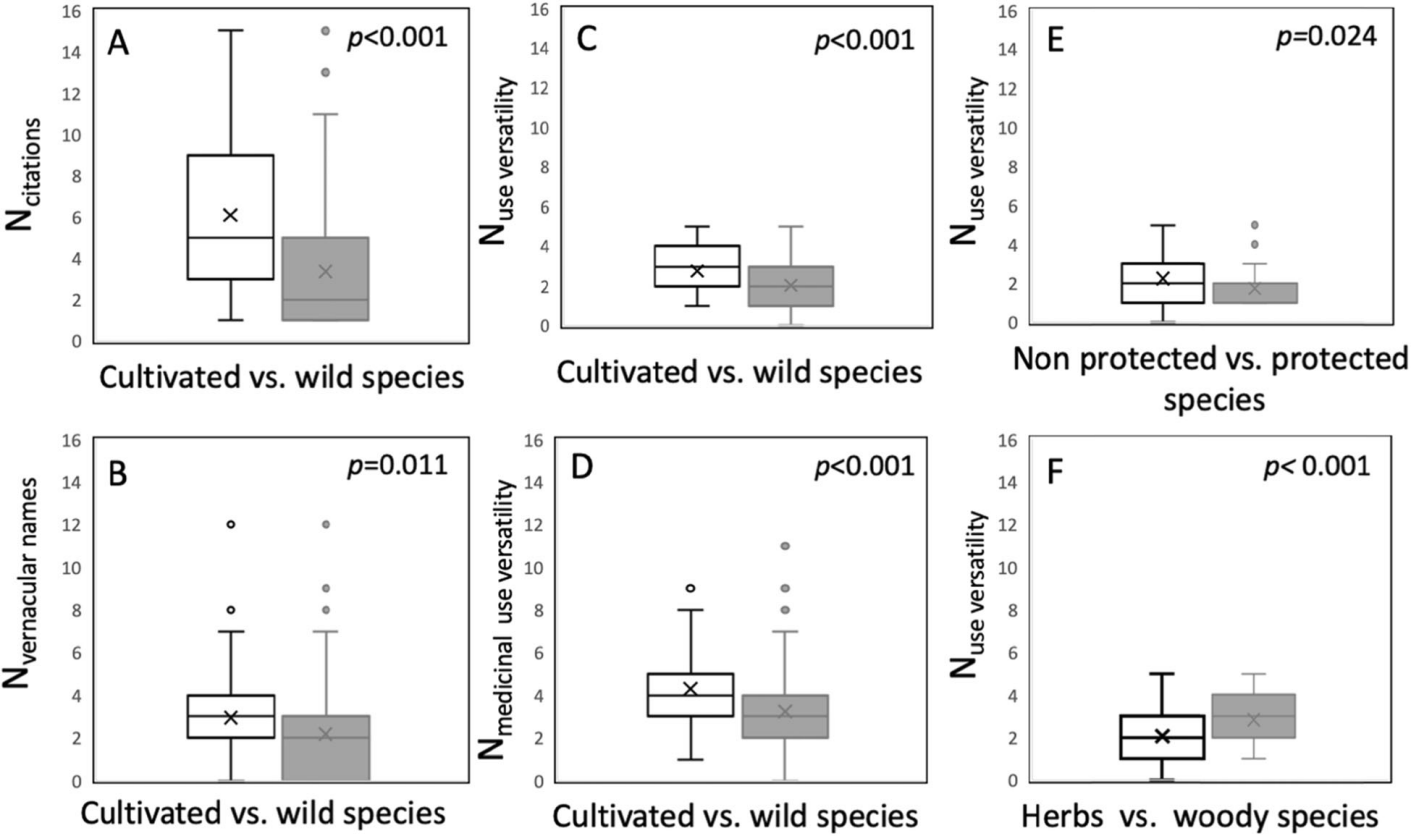
Comparison of cultivated versus wild medicinal species regarding the **a** number of citations in folk medicinal literature, **b** number of vernacular names, **c** overall use versatility, and **d** medicinal use versatility in South Tyrol. Number of overall use versatility comparing non protected and (partially) protected species (**e**) and herbs versus woody species (shrubs and trees; **f**). For results of the Kruskal-Wallis rank sum test, see text

Our analysis revealed that the use and subsequent abandonment of 17 species in South Tyrol is not linked to new pharmacological or phytochemical evidence on potential negative health effects or due to species conservation measures (protection or Red List status, Fig. 5). Moreover, there is limited scientific evidence on medicinal effects. Only 41% of the species, namely the most frequently cited species, have been explored by pharmacological studies in terms of their effects (Fig. 4).

Considering the most frequent families to which the medicinal plants belong (Asteraceae, Rosaceae, and Lamiaceae), our results were in high accordance with similar studies in European Alpine areas [3, 53–55]. The dominance of Asteraceae in local pharmacological literature worldwide has hitherto been emphasized (e.g., [68, 69]). With regard to the most frequently quoted species, there was a general agreement between our results and other studies from Northern Italy. In particular, the species *Achillea moschata*, *Achillea millefolium*, *Arnica montana*, *Urtica dioica*, and *Thymus pulegioides* were highly valued throughout all regions [3, 53–55, 70]. Interestingly, the third most cited plant, *Plantago lanceolata*, was used only rarely or not at all in other northern Italian regions. Instead, the closely related *P*. *major* is used, which, while morphologically distinct, has similar medicinal properties [3, 55, 70]. Also, the two frequently cited plants, *Equisetum arvense* and *Juniperus communis*, appeared to be of minor importance in other northern Italian regions.

We observed a high overall use versatility in South Tyrol (Fig. 3). As was reported in other alpine areas [53, 54, 70], in South Tyrol the most frequent therapeutic uses, and the uses that accounted for the highest number of plants, were for digestive, respiratory, and integumentary systems (Table 3).

The majority (59%) of native South Tyrolean medicinal plants remain understudied by medicine and pharmacology. However, considering the success rate (> 70%) of previously investigated plants (111) for which the traditional uses have been validated (79), we get an idea of the large unlocked phytotherapeutic and economic potential of as yet unexplored plants. In particular, the healing properties of the following largely unexplored plants should be analyzed, i.e., the highly quoted species *Peucedanum ostruthium* (wound healing, anti-inflammatory, digestive, and respiratory properties), *Juniperus communis* (antiseptic and for genitourinary problems), *Alchemilla* sp. and *Achillea* sp. (gynecology), *Allium ursinum* (cardiovascular, skin, digestion, and respiration), *Campanula rotundifolia* (borreliosis), *Dryopteris filix-mas* (antirheumatic and wound healing), *Euphrasia sp*. (antibacterial and anti-inflammatory for eye and respiration), *Geranium robertianum* (genitourinary, wound healing and infertility), *Larix decidua* (wound healing and disinfectant), *Pinus mugo* (expectorant), *Sempervivum tectorum* (wound healing), *Stachys sylvatica* (wound-healing and digestion), and *Veronica officinalis* (respiration, digestion, and musculoskeletal). A few almost exclusive uses were also found in the study area that, to our knowledge, have not been documented before in other Alpine areas. An example of an undocumented plant is of the *Geranium robertianum*, the flower and leaves of which are used in a tea to help with fertility issues [71]. Another is the use of *Botrychium lunaria* as an abortifacient [72].

Our results revealed a strong association between food and medicinal uses of the plants, including 128 aromatic or food related species (46.5% of all mentioned species) from over 50 families (Table 1). The most beneficial effects were on digestive, respiratory, skin, and general and unspecific disorders (Table 3). In fact, considering the increasing importance attributed to nutrition on human health maintenance, both therapeutic or prophylactic [73, 74], these plants could be of high relevance for the development of nutraceuticals [20]. This may be of particular interest to local sustainability projects that focus on local foods, eco-gastronomy, and organic farming. Examples include juice made from *Sambucus nigra* berries with antiviral and antimicrobial effects [75, 76], jam made out of *Rosa canina* fruits as a supplement of vitamin C [77], or *Thymus* sp. as spice with antibacterial effects [78]. Further research is needed to determine the nutritional and chemical compounds as well as safety for the commercialization of these exceptional food sources.

### Traditional medicinal plants for the conservation of nature and cultural heritage

For some years, there has been an emerging interest in the use of traditional ethnobotanical knowledge and plant resources [64]. While in the past, medicinal plants were mainly used for home consumption, the cultivation and use of medicinal plants has become a growing market niche in South Tyrol in the last few decades [51]. We recorded at least 21 farms where the main income source was herb production [79], and several gastronomy and hotel businesses that offered seasonal dishes or wellness and recreation programs based on traditional plants and practices [80]. In fact, the commercial use of herbal plants seems to offer numerous positive effects for the economic and ecological sustainability of South Tyrol, i.e., (i) the diversification of agricultural production, (ii) the maintenance of rural economies, (iii) the establishment of local value chains, (iv) the preservation of traditional land-use types (e.g., larch meadows or semi-arid grasslands; [81]) through extensive and ecologically oriented farming systems, (v) the maintenance of an important source of food supply for insects, and (vi) the preservation and revitalization of local identities, with a likely positive effect on biodiversity conservation.

Considering the positive market prospects for herbal products, with annual growth rates between 8 and 15% in Europe, North America, and Asia [82], traditional knowledge and plant resources offer a support for local economies. In this context, the various edible and medicinal plants identified in this study could provide opportunities for further diversification of mountain agriculture. Nevertheless, traditional medicinal plants still lack recognition as an economic factor in South Tyrol. Therefore, further research on agro-ecology, nutrition, bioactivity, and safety are highly important for the commercialization of tradition medicinal plants [21].

Many medicinal plants in the study area were threatened by anthropogenic and natural factors. Our analysis showed that 238 of the medicinal plant species (86%) were abundant, whereas 24 species (9%) were very rare and placed under the category of “conservation concern” on the regional Red List [29] (Table 2). Land-use changes, overexploitation, and climate change are considered to be the most serious threats to medicinal plants in Alpine regions [2, 19, 64]. Biodiversity in the Alps is closely linked to the interaction between the natural environment and traditional human practices [33]. The changes in social, touristic, and agricultural systems in the last decades led to a substantial conversion of land-use systems. This includes the intensification of land use in easily accessible areas [83] as well as the abandonment of traditional practices in remote areas that results in a decrease of species diversity and abundance [81, 84]. Consequently, along with the decrease in biodiversity, the abundance of medicinal plants is also affected [6]. In South Tyrol, the abandonment of alpine meadows and pastures along with the related expansion of areas of shrubs and trees has led to a decline of several heliophilous grassland species such as *Arnica montana* and *Centaurium erythraea* [85]. Moreover, many medicinal plants (e.g., *Carlina acaulis*, *Gentiana acaulis*, *Anemone vernalis*) that flourish in poor soils have been affected by increased nutrient input caused by fertilization [81].

Unsustainable exploitation of wild collected species is a well-known effect of booming markets with rising demands [64]. In addition, for South Tyrol, an increasing pressure on wild species has been reported by T. Wilhalm (pers. comm., Sept. 24, 2019). However, a closer look at the life forms and plant parts harvested reveals that not all species are equally affected by collection pressure. Root harvesting as a common practice in South Tyrol can be a severe threat to some rare medicinal plant species. Among root-harvested plants, we identified 6 particularly endangered species, three of which are under protection, two that are unprotected (*Dipsacus fullonum* and *Althaea officinalis*), and one species (*Eryngium campestre*) that is already extinct (Table 2). These threatened but unprotected plants should be re-considered and introduced into the South Tyrolean legislation.

Global warming and the associated upward migration of vegetation has become a major threat to specialized Alpine plants, particular those that inhabit the alpine-nivale altitudinal zones (> 2600 m a.s.l.) [22, 86]. Based on the GLORIA project data set, [22] concluded that for South Tyrol, with its highest peaks at almost 4000 m a.s.l., most plants can continue to invade higher elevations and, thus, the risk of extinction seems to be low. However, [22] identified two species (*Artemisia genipi* and *Primula glutinosa*) that are restricted to the upper Alpine zone and therefore might not be able to migrate further upward. Based on the same methodology, but with a larger data set, we identified at least 10 additional medicinal plant species that are restricted to the upper Alpine zone and therefore might be endangered by warming, at least locally (Table 4). In the foreseeable future, this could be the case in the Sella/Latemar region, for example, or the Texel group where the highest summits do not exceed 3200 m and the elevation distance between upper alpine and highest summit is less than 600 m a.s.l. On the other hand, however, some endangered medicinal plants such as the thermophilous species *Marrubium vulgare* may also benefit from warming.

## Conclusion

This study recorded the use of 275 traditional medicinal plants in South Tyrol. The values of EPI and EI show that ethnobotanical knowledge and plant diversity in the area were among the highest in Italy and the European Alps. Our results show a loss of local traditional knowledge and plants in the region, where over 85 of medicinal plants are listed in the regional Red List. On the other hand, the renewed interest in natural medicine has transformed the use of traditional medicinal plants into a new market niche for mountain agriculture in South Tyrol. Vulnerable but unprotected plant species should be reconsidered and introduced into the South Tyrolean legislation or addressed by ecosystem restoration measures. Furthermore, several plant species that are highly valued in local folk medicine remain understudied within medicine and pharmacology and could thus provide a starting point for further studies that may lead to the discovery of new molecules and opportunities for the diversification of mountain agriculture. The conservation and cultivation of traditional medicinal plants as well as the conservation and restoration of their habitats could provide new services for society and for land use and thus contribute to the population's wellbeing and ecologically sustainable development.

## Supplementary information

**Additional file 1.** Appendix A.

**Additional file 2.** Appendix B.

**Additional file 3.** Appendix C.

## Data Availability

The datasets used and/or analyzed during the current study are available in the appendices of the study. Further information is available from the corresponding author on reasonable request.

## References

[1] Bussmann RW. Ethnobotany of the Samburu of Mt. Nyiru, South Turkana, Kenya. J Ethnobiol Ethnomed. 2006;2(35). 10.1186/1746-4269-2-35 PMC1570449 16956401

[2] Salick J, Zhendong F, Byg A (2009). Eastern Himalayan alpine plant ecology, Tibetan ethnobotany, and climate change. Glob Environ Chang.

[3] Vitalini S, Iriti M, Puricelli C (2013). Traditional knowledge on medicinal and food plants used in Val san Giacomo (Sondrio, Italy) - an alpine ethnobotanical study. J Ethnopharmacol.

[4] Joshi AR, Joshi K (2000). Indigenous knowledge and uses of medicinal plants by local communities of the Kali Gandaki watershed area, Nepal. J Ethnopharmacol.

[5] Patwardhan B (2005). Ethnopharmacology and drug discovery. J Ethnopharmacol.

[6] Neergheen-Bhujun V, Awan AT, Baran Y (2017). Biodiversity, drug discovery, and the future of global health: introducing the biodiversity to biomedicine consortium, a call to action. J Glob Health.

[7] Chapin FS, Zavaleta ES, Eviner VT (2000). Consequences of changing biodiversity. Nature.

[8] Butchart SHM, Walpole M, Collen B (2010). Global biodiversity: indicators of recent declines. Science.

[9] Thomas CD, Cameron A, Green RE (2004). Extinction risk from climate change. Nature.

[10] Vittoz P, Cherix D, Gonseth Y (2013). Climate change impacts on biodiversity in Switzerland: a review. J Nat Conserv.

[11] Pertoldi C, Bach LA (2007). Evolutionary aspects of climate-induced changes and the need for multidisciplinarity. J Therm Biol.

[12] Pimm SL, Jenkins CN, Abell R (2014). The biodiversity of species and their rates of extinction, distribution, and protection. Science.

[13] Sen T, Samanta SK (2014). Medicinal plants, human health and biodiversity: a broad review. Adv Biochem Eng Biotechnol.

[14] UNCED (2018). Convention on biological diversity.

[15] UNESCO (2002). The universal declaration on cultural diversity. United Nations Educational, Scientific and Cultural Organization, Paris. France..

[16] Berkes F. Sacred ecology: Routledge; 2017.

[17] Niedrist G, Tasser E, Lüth C (2009). Plant diversity declines with recent land use changes in European Alps. Plant Ecol.

[18] Tattoni C, Ianni E, Geneletti D (2017). Landscape changes, traditional ecological knowledge and future scenarios in the Alps: a holistic ecological approach. Sci Total Environ.

[19] Pieroni A, Giusti ME (2009). Alpine ethnobotany in Italy: traditional knowledge of gastronomic and medicinal plants among the Occitans of the upper Varaita valley. Piedmont J Ethnobiol Ethnomed.

[20] Abbet C, Mayor R, Roguet D (2014). Ethnobotanical survey on wild alpine food plants in lower and Central Valais (Switzerland). J Ethnopharmacol.

[21] Pinela J, Carvalho AM, Ferreira ICFR (2017). Wild edible plants: nutritional and toxicological characteristics, retrieval strategies and importance for today’s society. Food Chem Toxicol.

[22] Grabherr G (2009). Biodiversity in the high ranges of the Alps: ethnobotanical and climate change perspectives. Glob Environ Chang.

[23] Pleszczyńska M, Lemieszek MK, Siwulski M, et al. Fomitopsis betulina (formerly Piptoporus betulinus): the Iceman’s polypore fungus with modern biotechnological potential. World J Microbiol Biotechnol. 2017;33. 10.1007/s11274-017-2247-0 PMC5380686 28378220

[24] European Soil Data Centre (ESDAC) (2012). European Commission. Joint Research Centre.

[25] ASTAT (2018). *Stati*stisches Jahrbuch Für Südtirol 2018.

[26] Karte PT, Südtirols DAV. Autonome Provinz Bozen-Südtirol - Amt für Naturparke. Wien: Naturschutz Und Landscaftspflege; 1991.

[27] Oeschger H, Messerli B, Svilar M (1980). Das Klima von Tirol - Südtirol - Belluno.

[28] Wilhalm T, Gutermann W, Niklfeld H (2006). Katalog Der Gefässpflanzen Südtirols. Auflage: 1.

[29] Wilhalm T. Hilpold. Rote Liste der gefährdeten Gefäßpflanzen Südtirols. Gredleriana. 2006:115–98.

[30] Zinn DL (2018). Migrants as metaphor.

[31] Pickl-Herk W. Volksmedizinische Anwendung von Arzneipflanzen im Norden Südtirols. Unpubl Diploma Thesis Univ Wien. 1995;295.

[32] Eichinger L (2002). South Tyrol: German and italian in a changing world. J Multiling Multicult Dev.

[33] Chemini C, Rizzoli A (2003). Land use change and biodiversity conservation in the Alps. J Mt Ecol.

[34] Shamseer L, Moher D, Clarke M, et al. Preferred reporting items for systematic review and meta-analysis protocols (prisma-p) 2015: elaboration and explanation. BMJ. 2015;349. 10.1136/bmj.g7647 25555855

[35] Mayer M, Zbinden M, Vogl CR (2017). Swiss ethnoveterinary knowledge on medicinal plants - a within-country comparison of Italian speaking regions with north-western German speaking regions. J Ethnobiol Ethnomed.

[36] Stucki K, Cero MD, Vogl CR (2019). Ethnoveterinary contemporary knowledge of farmers in pre-alpine and alpine regions of the Swiss cantons of Bern and Lucerne compared to ancient and recent literature – is there a tradition?. J Ethnopharmacol.

[37] Owen DJ, Fang M-LE (2003). Information-seeking behavior in complementary and alternative medicine (CAM): an online survey of faculty at a health sciences campus. J Med Libr Assoc.

[38] Südtirol N. FloraFaunaSüdtirol. Das Portal zur Verbreitung von Tier- und Pflanzenarten in Südtirol. 2019.

[39] Nimis PL, Martellos S (2017). ITALIC 5.0 - the information system on Italian lichens. Version 5.

[40] Curti PAMINT. Associazione Micologica Italiana Naturalistica. Telematica. 2019.

[41] The Plant List (2013). Version 1.1. Published on the internet.

[42] Cunningham AB. Applied ethnobotany : people, wild plant use, and conservation: Earthscan; 2001.

[43] Berlin B. Ethnobiological classification : principles of categorization of plants and animals in traditional societies: Princeton University Press; 1992.

[44] ICPC-2. International Classification of Primary Care second ed.

[45] Staub PO, Geck MS, Weckerle CS (2015). Classifying diseases and remedies in ethnomedicine and ethnopharmacology. J Ethnopharmacol.

[46] Bundesinstitut für Arzneimittel und Medizinprodukte (2002). Liste der Monographien der Kommission E (Phytotherapie). Die im Bundesanzeiger veröffentlicht Sind.

[47] European Union monographs and list entries | European Medicines Agency.

[48] Menendez-Baceta G, Aceituno-Mata L, Molina M (2014). Medicinal plants traditionally used in the northwest of the Basque Country (Biscay and Alava), Iberian Peninsula. J Ethnopharmacol.

[49] Portères R (1970). Ethnobotanique Générale.

[50] Bonet MÀ, Parada M, Selga A (1999). Studies on pharmaceutical ethnobotany in the regions of L’Alt Empordà and les Guilleries (Catalonia, Iberian Peninsula). J Ethnopharmacol.

[51] Schunko C, Lechthaler S, Vogl CR. Conceptualising the factors that influence the commercialisation of non-timber forest products: the case of wild plant gathering by organic herb farmers in South Tyrol (Italy). Sustainability. 2019;11.

[52] Fischer MA, Gottschlich G. Exkursionsflora Für Österreich, Liechtenstein Und Südtirol Bestimmungsbuch Für Alle in Der Republik Österreich, in Der Autonomen Provinz Bozen/Südtirol (Italien) Und Im Fürstentum Liechtenstein Wildwachsenden Gefäßpflanzen (Farnpflanzen und Samenp. OÖ*)* Landesmuseen, 2005.

[53] Cornara L, La Rocca A, Terrizzano L (2014). Ethnobotanical and phytomedical knowledge in the North-Western Ligurian Alps. J Ethnopharmacol.

[54] Vitalini S, Puricelli C, Mikerezi I (2015). Plants, people and traditions: Ethnobotanical survey in the Lombard Stelvio National Park and neighbouring areas (Central Alps, Italy). J Ethnopharmacol.

[55] Dei Cas L, Pugni F, Fico G (2015). Tradition of use on medicinal species in Valfurva (Sondrio, Italy). J Ethnopharmacol.

[56] Tali BA, Khuroo AA, Nawchoo IA (2019). Prioritizing conservation of medicinal flora in the Himalayan biodiversity hotspot: an integrated ecological and socioeconomic approach. Environ Conserv.

[57] Assefa A, Bahiru A (2018). Ethnoveterinary botanical survey of medicinal plants in Abergelle, Sekota and Lalibela districts of Amhara region, northern Ethiopia. J Ethnopharmacol.

[58] Gao L, Wei N, Yang G, et al. Ethnomedicine study on traditional medicinal plants in the Wuliang Mountains of Jingdong, Yunnan, China. J Ethnobiol Ethnomed. 2019;15. 10.1186/s13002-019-0316-1 PMC6699132 31426826

[59] Ferrier J, Saciragic L, Trakić S, et al. An ethnobotany of the Lukomir highlanders of Bosnia & Herzegovina. J Ethnobiol Ethnomed. 2015;11. 10.1186/s13002-015-0068-5 PMC4658798 26607753

[60] Guarrera P, Lucchese F, Medori S (2008). Ethnophytotherapeutical research in the high Molise region (central-southern Italy). J Ethnobiol Ethnomed.

[61] Lokar LC, Poldini L (1988). Herbal remedies in the traditional medicine of the Venezia Giulia region (north East Italy). J Ethnopharmacol.

[62] Mustafa B, Hajdari A, Krasniqi F (2012). Medical ethnobotany of the Albanian Alps in Kosovo. J Ethnobiol Ethnomed.

[63] Wegmann U. Ethnobotanik im Prättigau. Medizinalpflanzen - Nutzung und Wissen. Master-Thesis, Univ Zürich, Switzerland 2013.

[64] Schippmann U, Leaman DJ, Cunningham AB (2002). Impact of cultivation and gathering of medicinal plants on biodiversity: global trends and issues.

[65] Guarrera PM, Lucia LM. Ethnobotanical remarks on central and southern Italy, vol. 3; 2007. 23. 10.1186/1746-4269-3-23 PMC1906747 17537240

[66] Meraner R, Oberhofer M. Dialekte und Hochsprache in der Schule; Arbeitskreis Südtiroler Mittelschullehrer. Hrsg. Von Kurt Egger, Bozen, 1982. 1982.

[67] Petrovska BB (2012). Historical review of medicinal plants’ usage. Pharmacogn Rev.

[68] Heinrich M, Robles M, West JE (1998). Ethnopharmacology of Mexican Asteraceae (Compositae). Annu Rev Pharmacol Toxicol.

[69] Saslis-Lagoudakis CH, Williamson EM, Savolainen V (2011). Cross-cultural comparison of three medicinal floras and implications for bioprospecting strategies. J Ethnopharmacol.

[70] Vitalini S, Tomè F, Fico G (2009). Traditional uses of medicinal plants in Valvestino (Italy). J Ethnopharmacol.

[71] Hochgruber G. Heilkräuter - Die Apotheke der Natur Alternative Heilmethoden Nach Gottfried Hochgruber., 2018.

[72] Achmüller A. Teufelskraut, Bauchwehblüml, Wurmtod: Das Kräuterwissen Südtirols: Mythologie, Volksmedizin und Wissenschaftliche Erkenntnisse. Edition Raetia, 2012.

[73] Etkin NL (1996). Medicinal cuisines: diet and ethopharmacology. Int J Pharmacogn.

[74] Tapsell LC, Neale EP, Satija A (2016). Foods, nutrients, and dietary patterns: interconnections and implications for dietary guidelines. Adv Nutr.

[75] Chen C, Zuckerman DM, Brantley S (2014). Sambucus nigra extracts inhibit infectious bronchitis virus at an early point during replication. BMC Vet Res.

[76] Krawitz C, Mraheil MA, Stein M (2011). Inhibitory activity of a standardized elderberry liquid extract against clinically-relevant human respiratory bacterial pathogens and influenza a and B viruses. BMC Complement Altern Med.

[77] Mihoc M, Mihai C (2008). Ascorbic acid content in extractive aqueous solutions of Rosa canina L. Fruits Agric Conspec Sci.

[78] Rasooli I, Mirmostafa SA (2002). Antibacterial properties of Thymus pubescens and Thymus serpyllum essential oils. Fitoterapia.

[79] Vereinigung Südtiroler Kräuteranbauer.

[80] Karnutsch V. Innovation und bäuerliche tradition am Beispiel der Südtiroler Heubäder. Diploma Thesis, Free Univ Bolzano. 2004;126.

[81] Zerbe S. Renaturierung von Ökosystemen Im Spannungsfeld von Mensch und Umwelt: Ein Interdisziplinäres Fachbuch. SPRINGER, 2019.

[82] Verma S, Singh S (2008). Current and future status of herbal medicines. Vet World.

[83] Grabherr G (2010). Biodiversitätsverlust durch moderne Hochlagen-Landwirtschaft. Jahrb des Vereins zum Schutz der Bergwelt.

[84] Tasser E, Tappeiner U, löschen S, et al. Impact of land use changes on mountain vegetation. Appl Veg Sci. 2002;5:173–84.

[85] Michler B, Rotar I, Pacurar F et al. Arnica montana, an endangered species and a traditional medicinal plant: the biodiversity and productivity of its typical grasslands habitats. Integrating Efficient Grassland Farming and Biodiversity. Vol 10. Proceedings of EGF, Estonia, 2005, 336–40.

[86] Chelli S, Wellstein C, Campetella G (2017). Climate change response of vegetation across climatic zones in Italy. Clim Res.

