# Supplementary material for: Traditional medicinal plants in South Tyrol (northern Italy, southern Alps): biodiversity and use
Source: J Ethnobiol Ethnomed. 2020 Nov 26;16:74. doi: 10.1186/s13002-020-00419-8 (PMC7690129; doi:10.1186/s13002-020-00419-8)
Supplement: Supplementary file 1 — Additional file 1. Appendix A. [file 13002_2020_419_MOESM1_ESM.docx]

1. Based on systematic literature review of web of knowledge using the keywords “medicinal,” “medical,” or “phytomedicine” and “alpine species” in “title” or “topic” following the PRISMA guidelines we identified 21 references:

| Approach | Reference |
| --- | --- |
| Using Data from Interviews | [1] (Lower and Central Valais, Switzerland); [2] (Trentino, Italy); [3] (Albanian Alps, Kosovo, Serbia); [4] (Central Alps, Italy); [5] (Piedmont, Italy); [6] (Lombardia, Italy); [7] (Switzerland); [8] (Bern, Lucerne, Switzerland); [9] (Slovenia); [10] (Ligurian Alps, Italy) |
| Experimental approaches on selected species | [11]; [12]; [13]; [14]; [15]; [16]; [17]; [18] |
| Land use change and medicinal species | [19]; [20] |
| Review on selected species | [21] |

In addition, we included the following papers on wild and cultivated plants as food and medicine used by Alpine minorities [22–24].

Body of literature (only german speaking literature) with information on plant species used for medicinal purposes in South Tyrol; type of reference with C = cultural literature, P = prescriptions book, S = scientific manuscript; N = number of medicinal plant species stated, originality of information with O = original, S = secondary, OS = original & secondary

| Reference | Type | N | Originality |
| --- | --- | --- | --- |
| [25] | P | 71 | OS |
| [26] | P | 44 | OS |
| [27] | C | 66 | S |
| [28] | P | 38 | S |
| [29] | P | 98 | OS |
| [30] | P | 67 | OS |
| [31] | C | 83 | O |
| [32] | P | 83 | OS |
| [33] | C | 51 | O |
| [34] | P | 112 | OS |
| [35] | S | 155 | O |
| [36] | P | 100 | OS |
| [37] | C | 90 | O |
| [38] | C | 18 | S |
| [39] | C | 82 | O |
| [40] | P | 38 | OS |
| [41] | C | 31 | O |
| [42] | S | 1 | OS |

Reference list Appendix A

1. Abbet C, Mayor R, Roguet D *et al*. Ethnobotanical survey on wild alpine food plants in Lower and Central Valais (Switzerland). J Ethnopharmacol 2014;151:624–34

2. Vitalini S, Iriti M, Puricelli C *et al*. Traditional knowledge on medicinal and food plants used in Val San Giacomo (Sondrio, Italy) - An alpine ethnobotanical study. J Ethnopharmacol 2013;145:517–29

3. Mustafa B, Hajdari A, Krasniqi F *et al*. Medical ethnobotany of the Albanian Alps in Kosovo. J Ethnobiol Ethnomed 2012;8:1–14

4. Grabherr G. Biodiversity in the high ranges of the Alps: Ethnobotanical and climate change perspectives. Glob Environ Chang 2009;19:167–72

5. Pieroni A, Giusti ME. Alpine ethnobotany in Italy: Traditional knowledge of gastronomic and medicinal plants among the Occitans of the upper Varaita valley, Piedmont. J Ethnobiol Ethnomed 2009;5:1–13

6. Vitalini S, Puricelli C, Mikerezi I *et al*. Plants, people and traditions: Ethnobotanical survey in the Lombard Stelvio National Park and neighbouring areas (Central Alps, Italy). J Ethnopharmacol 2015;173:435–58

7. Mayer M, Zbinden M, Vogl CR *et al*. Swiss ethnoveterinary knowledge on medicinal plants - a within-country comparison of Italian speaking regions with north-western German speaking regions. J Ethnobiol Ethnomed 2017;13:1

8. Stucki K, Cero MD, Vogl CR *et al*. Ethnoveterinary contemporary knowledge of farmers in pre-alpine and alpine regions of the Swiss cantons of Bern and Lucerne compared to ancient and recent literature – Is there a tradition? J Ethnopharmacol 2019;234:225–44

9. Povšnar M, Koželj G, Kreft S *et al*. Rare tradition of the folk medicinal use of Aconitum spp. is kept alive in Solčavsko, Slovenia. J Ethnobiol Ethnomed 2017;13

10. Cornara L, La Rocca A, Terrizzano L *et al*. Ethnobotanical and phytomedical knowledge in the North-Western Ligurian Alps. J Ethnopharmacol 2014;155:463–84

11. Zidorn C. Altitudinal variation of secondary metabolites in flowering heads of the Asteraceae: Trends and causes. Phytochem Rev 2010;9:197–203

12. Hartwich M. The importance of immunological studies on Rhodiola rosea in the new effective and safe herbal drug discovery. Cent J Immunol 2010;35:263–6

13. Tomczyk M, Latté KP. Potentilla-A review of its phytochemical and pharmacological profile. J Ethnopharmacol 2009;122:184–204

14. Marzocco S, Adesso S, Alilou M *et al*. Anti-inflammatory and anti-oxidant potential of the root extract and constituents of doronicum austriacum. Molecules 2017;22

15. Lütken H, Meropi-Antypa N, Kemp O *et al*. Hairy root cultures of Rhodiola rosea to increase valuable bioactive compounds. *Production of Plant Derived Natural Compounds through Hairy Root Culture*. Springer International Publishing, 2017, 65–88.

16. Caser M, Victorino ÍMM, Demasi S *et al*. Saffron Cultivation in Marginal Alpine Environments: How AMF Inoculation Modulates Yield and Bioactive Compounds. Agronomy 2018;9:12

17. Vitalini S, Madeo M, Tava A *et al*. Chemical profile, antioxidant and antibacterial activities of achillea moschata wulfen, an endemic species from the alps. Molecules 2016;21

18. Aiello N, Bontempo R, Vender C *et al*. Morphological and qualitative characteristics of Rhodiola rosea L. wild populations of Trentino, Italy. J Med spice plants 2013;18:41–5

19. Maseyk FJF, Demeter L, Csergő AM *et al*. Effect of management on natural capital stocks underlying ecosystem service provision: a ‘provider group’ approach. Biodivers Conserv 2017;26:3289–305

20. Mardari C, Birsan C, Stefanache C *et al*. Population structure and habitat characteristics of Arnica montana L. in the NE Carpathians (Romania). Tuexenia 2019:401–21

21. Tauchen J, Kokoska L. The chemistry and pharmacology of Edelweiss: a review. Phytochem Rev 2017;16:295–308

22. Obón C, Rivera D, Alcaraz F. Wild and cultivated plants used as food and medicine by the mòcheni ethnic minority in the Alps. Acta Hortic 2012;955:113–8

23. Rivera D, Alcaraz F, Obón C. Wild and cultivated plants used as food and medicine by the cimbrian ethnic minority in the Alps. Acta Hortic 2012;955:31–40

24. Membretti A, Viazzo PP. Negotiating the Mountains. Foreign Immigration and Cultural Change in the Italian Alps. Martor Rev d’Anthropologie du Musée du Paysan Roum 2017:93–107

25. Achmüller A. *Teufelskraut*, *Bauchwehblüml*, *Wurmtod: Das Kräuterwissen Südtirols: Mythologie*, *Volksmedizin Und Wissenschaftliche Erkenntnisse*. Edition Raetia, 2012

26. Achmüller A. *Die Alpen-Apotheke : Hausmittel Zum Selbermachen*., 2015

27. Canestrini M. *Bauerngärten in Südtirol*. Folio, 2012

28. Dander C, Kompatscher A. *Garten Der Gesundheit: Heilkräuter in Südtirol: Wissenwertes Aus Mythologie*, *Volksglauben Und Medizin*. Tappeiner, 1991

29. Hager I, SchÖnweger A, HÖnigschmid A *et al*. *Die Kraft Der Südtiroler Kräuter Nutzen 350 Rezepte Für Wohlbefinden*, *SchÖnheit*, *Küche*, *Haus Und Garten*., 2014

30. Hochgruber G. *Heilkräuter - Die Apotheke Der Natur Alternative Heilmethoden Nach Gottfried Hochgruber*., 2018

31. Marsoner-Staffler Z, Schwienbacher M, Edition Raetia GmbH. *Treiner Rosa Hausmittel Einer Kräuterfrau*., 2017

32. Mayr C, Niederegger O. *Heilpflanzen Der Alpen: Gesundheit Aus Der Natur von A Bis Z*. Tyrolia, 2006

33. Moroder MM. *Heilpflanzen in Den Dolomiten: Volksmedizin*, *Volksglaube*, *Volksaberglaube*. Athesia Touristik, 2003

34. Niederegger O, Mayr C. *Hausbuch Der Südtiroler Heilkräuter: Gesundheit Aus Der Natur*. Athesia, 2005

35. Pickl-Herk W. *Volksmedizinische Anwendung von Arzneipflanzen Im Norden Südtirols*. Unpubl Diploma Thesis Univ Wien. 1995;295.

36. Plaikner F, Mayr C. *Hausbuch Der Südtiroler Heilpflanzen*. Athesia, 1988

37. SchÖnweger A, Hager von Strobele I, HÖnigschmid A. *Südtiroler Kräuterfrauen Ihr Leben*, *Ihr Heilwissen*, *Ihre Rezepte*. LÖwenzahn Verlag, 2014

38. Schwienbacher B. *Wenn KÖrper Und Seele Zueinander Finden Altes Heil- Und Lebenswissen Aus Den Südtiroler Bergen*. Integral, 2015

39. Asche R, Schulze ED. *Die Ragginer : 200 Jahre Volksmedizin in Südtirol*. Munchen : Dr. Friedrich Pfeil, 1996

40. Somvi D. *Kräuterwanderungen in Südtirol Kräuter Für Hausapotheke*, *Küche Und Wohlbefinden*. Tappeiner Verlag, 2015

41. Waldboth J, Schrott B. *Für Viech Und Leit: Bäuerliche Volksmedizin Aus Dem Eisacktal*., 2011

42. Kiem J. Ein Tamariskenvorkommen im Sarntal. BerBayerBotGes 1992:139–43
