# Supplementary material for: Traditional medicinal plants in South Tyrol (northern Italy, southern Alps): biodiversity and use
Source: J Ethnobiol Ethnomed. 2020 Nov 26;16:74. doi: 10.1186/s13002-020-00419-8 (PMC7690129; doi:10.1186/s13002-020-00419-8)
Supplement: Supplementary file 2 — Additional file 2. Appendix B. [file 13002_2020_419_MOESM2_ESM.docx]

Appendix B

Local vernacular names of the german linguistic group for traditional medicinal plants used in the region of South Tyrol

| Plant species | Count | Vernacular names |
| --- | --- | --- |
| *Abies alba* | 1 | Taxn |
| *Achillea atrata* | 3 | Ziegenkräutl, Geschwärtzte Scharfgarbe, Edelraute |
| *Achillea clavennae* | 4 | Weißer Speik, Bergwehmut, Kührauten, Steinrauten |
| *Achillea millefolium* | 17 | Bauchwehbluiml, Blutstillkrutt, Boanfraßkräutl, Bucklkräutl, Dochal, Feldgarbe, Fochal, Frauendank, Frauenkräutl, Gochal, Haboraut, Heil aller Schäden, Herrgottsbucklkräutl, Kochal, Scharfgabenkräutl, Tochaln, Touchal |
| *Achillea moschata* | 4 | Jochkiefer, Jochtochaln, Kleine Scharfgarbe, Iva |
| *Aconitum napellus* | 3 | Sturmhut, Teufelwurz, Nonnenhaubal |
| *Adiantum capillus-veneris* | 4 | Wiederton, Mondfarn, Frauenhaar, Friggagras |
| *Aegopodium podagraria* | 6 | Girsch, Podagrakrutt, Huhnschinken, Ziegenfuß, Dreiblattl, Zipperleinkräutl |
| *Aesculus hippocastanum* | 1 | Wilde Köstn |
| *Agrimonia eupatoria* | 2 | Heil aller Welt, König aller Kräuter |
| *Ajuga reptans* | 0 |  |
| *Alchemilla alpina* | 3 | Silbermantel, Marienmantelkrutt, Taumantelkräutl |
| *Alchemilla xanthochlora* | 18 | Alchemistenkraut, Frauenhilf, Frauenmantele, Lägerkrut, Mantelekrut, Marienmantele, Marienmantelkrutt, Muttergotteskraut, Schienaukraut, Silbermantele, Taubercherl, Taubrech, Taumentele, Taumantelkrutt, Zinaukrut, Zinauplattl |
| *Alliaria petiolata* | 0 |  |
| *Allium schoenoprasum* | 2 | Schnitl, Schnittloab |
| *Allium ursinum* | 5 | Hexenzweifel, Wrumlauch, Wilder Knoblauch, Waldknoblauch, Zigeunerzweibel |
| *Allium victorialis* | 2 | Bergknoblauch, Lahnwurzn |
| *Althaea officinalis* | 4 | Eibisch, Heilwurzn, Ibisch, Altheenwurzn |
| *Anacamptis morio* | 0 |  |
| *Anemone vernalis* | 2 | Osterglocke, Pelzanemone |
| *Angelica sylvestris* | 0 |  |
| *Antennaria dioica* | 3 | Himmelfahrtsbluiml, Immorrtelle, Ewige Blume |
| *Anthriscus sylvestris* | 1 | Kerbel |
| *Anthyllis vulneraria* | 3 | Apothekerlklee, Katzenbratzerl, Bärenpratzen |
| *Aquilegia einseleana* | 0 |  |
| *Arabis petiolata* | 0 |  |
| *Arctium lappa* | 1 | Picker |
| *Arctostaphylos uva-ursi* | 7 | Mehlgranten, Wilde Grante, Ruschbeer, Wilde Preiselbeer, Rauschhorterer, Rossgranten , Ruschgranten |
| *Arnica montana* | 18 | Bergwohlverleih, Bergwurz, Donnerblume, Engenkrutt, Fallkrutt, Gamsblüml, Gamswurz, Kathereinkraut, Kraftrose, Kraftwurzn, Krieger, Mädebluiml, Ogsnbluiml, Wohlverleih, Wolfswurzn, Wolfsziesel, Wundkrutt |
| *Artemisia absinthium* | 9 | Abraut, Absinth, Bermand, Girtelkräutl, Johannisgurt, Magenkrutt, Permat, Schossmaltern, Wurmtod |
| *Artemisia mutellina* | 3 | Goldner Oberraut, Keesraute, Kleiner Baifuß |
| *Artemisia vulgaris* | 5 | Wilder Wehrmut, Gewürzbeifuß, Abraut, Machtwurzn, Sonnenwendkräutl |
| *Asparagus officinalis* | 0 |  |
| *Asplenium septentrionale* | 3 | Harngras, Haargras, Seifenfarn |
| *Athamanta cretensis* | 3 | Mohrenkümmel, Hirschwurzn, Bärenwurz |
| *Atropa bella-donna* | 0 |  |
| *Auricularia auricula-judae* | 2 | Judasohr, Ohrlappenpilz |
| *Bellis perennis* | 6 | Monatlan, Maßliebchen, Tausendschön, Sonnentürchen, Mutterblüml, Marienbluimel |
| *Berberis vulgaris* | 4 | Brumlstaude, Sauerdorn, Essigdorn, Bummelbeer |
| *Betonica officinalis* | 2 | Heil-Ziest, Zehrkräutl |
| *Betula pendula* | 4 | Weißbirke, Warzenbirke, Besenbaum, Hoarbirke |
| *Biscutella laevigata* | 1 | Brillensenf |
| *Borago officinalis* | 0 |  |
| *Botrychium lunaria* | 3 | Bettseichkrutt, Peterschlüssel, Hurenkräutl |
| *Brassica rapa* | 2 | Herbstrübe, Rübenkräutl |
| *Calluna vulgaris* | 4 | Horderer, Hiedekräutl, Erika, Schneeheide |
| *Campanula patula* | 2 | Glogu, Gras-Glockenblüml |
| *Campanula rotundifolia* | 2 | Gloguu, Glockenbluiml |
| *Capsella bursa-pastoris* | 4 | Taschlgras, Beutelschneiderkräu,l Seckelkrutt, Taschnickerkräutl |
| *Carlina acaulis* | 8 | Einhaken, Wetterdistel, Silberdistel, Karlsdistel, Mareindistel, Roßwurz, Karwendel, Eberwurzn |
| *Carum carvi* | 4 | Brotkümmel, Carvensamen, Garbe, Wiesenkümmel |
| *Castanea sativa* | 2 | Kesten, Riggl |
| *Centaurium erythraea* | 3 | Allerweltsheil, Erdgalle, Fieberkräutl |
| *Cerastium fontanum* | 0 |  |
| *Cetraria islandica* | 12 | Goastraubn, Halswehkrutt, Inschländische Flechte, Isere, Jochrispen, Lungenkraut, Lungenmoos, Misere, Rispal, Rispelreaspelkraut, Rispen, Ruspe |
| *Chelidonium majus* | 3 | Schwalbenkraut, Warzenkrutt, Schellwurzn |
| *Chenopodium bonus-henricus* | 2 | Goasenfuß, Wilder Spinat |
| *Cichorium intybus* | 3 | Wögwart, Wegleuchte, Feld-Zichorie |
| *Clinopodium alpinum* | 3 | Steinpolei, Bergthymian, Basilienthymian |
| *Corylus avellana* | 2 | Haselstaude, Hasele |
| *Crataegus monogyna* | 7 | Mehlbeer, Hagedorn, Zundorn, Schlafdorn, Muttergottesepfalan, Fraudenpfalan, Muttergottesbirlan |
| *Cyanus segetum* | 3 | Blaumützen, Kaiserblume, Kreuzblume |
| *Cyclamen purpurascens* | 3 | Erdscheibe, Kreuzwehkräutl, Guasrubn |
| *Daucus carota* | 2 | Riabe, Wild Rüabli |
| *Dentaria enneaphyllos* | 3 | Sanigl, Dreyblettrich, Zahnkräutl |
| *Diplotaxis tenuifolia* | 1 | Wiesn Rukula |
| *Dipsacus fullonum* | 0 |  |
| *Drosera rotundifolia* | 0 |  |
| *Dryas octopetala* | 4 | Wildes Weibal, Welsches Silberkräutl, Bergramanderlein |
| *Dryopteris filix-mas* | 10 | Farnkräutl, Fünffingerwurzn, Hennpfarfl, Johannishand, Labassen, Männerfarn, Otterkrutt, Pfofel, Wanzenwurz, Wurmblattl |
| *Elymus repens* | 1 | Schließgras |
| *Epilobium angustifolium* | 1 | Waldrosen |
| *Epilobium montanum* | 0 |  |
| *Epilobium palustre* | 0 |  |
| *Epilobium parviflorum* | 1 |  |
| *Equisetum arvense* | 4 | Zinnkräutl, Ackerschachtelhalm, Katzenschwanz, Katzenschweif |
| *Equisetum palustre* | 1 | Katzenschwanz |
| *Equisetum pratense* | 3 | Hainschachtelhalm, Zinnkraut, Katzenschwanz |
| *Erica carnea* | 1 | Schneeheide |
| *Erigeron alpinus* | 3 | Cespita, Flöhkräutl, Zauberwurzn |
| *Eriophorum angustifolium* | 2 | Wollgras, Jachwolle |
| *Eryngium amethystinum* | 0 |  |
| *Eryngium campestre* | 0 |  |
| *Euphrasia alpina* | 0 |  |
| *Euphrasia minima* | 0 |  |
| *Euphrasia officinalis* | 10 | Augenbluiml, Hirnkrutt, Hörbes gregg'n, Magentrost, Milchdieb, Milchraber, Milchschelm, Ruhrkraut, Spöttich, Zigeunerkräutl |
| *Euphrasia rostkoviana* | 3 | Milchdieb, Augenblume, Magenstrost |
| *Euphrasia stricta* | 0 |  |
| *Euphrasia versicolor* | 0 |  |
| *Fagus sylvatica* | 0 |  |
| *Filipendula ulmaria* | 5 | Wiesengeißbart, Geißbart, Krampfkräutl, Bienenkräutl, Wiesenkönigin |
| *Fomitopsis betulina* | 0 |  |
| *Fomitopsis officinalis* | 0 |  |
| *Fragaria vesca* | 1 | Monatserdbeere |
| *Fragaria viridis* | 1 | Monatserdbeere |
| *Fraxinus excelsior* | 2 | Goaßbaum, Schwindholz |
| *Fraxinus ornus* | 2 | Manna-Esche, Manna |
| *Fumaria officinalis* | 3 | Elfenrauch, Grindkräutl, Kratzheil |
| *Galega officinalis* | 2 | Bockskraut, Goasklee |
| *Galeopsis pubescens* | 0 |  |
| *Galium aparine* | 0 |  |
| *Galium mollugo* | 2 | Wiesenlabkrutt, Weißes Labkräutl |
| *Galium odoratum* | 2 | Herzfreund der Venus, Waldmännlein |
| *Galium verum* | 2 | Echtes Labkraut, Gelbes Labkräutl |
| *Genista tinctoria* | 0 |  |
| *Gentiana acaulis* | 3 | Blauer Enzian, Kuckucksblüml, Guckhantschen |
| *Gentiana lutea* | 7 | Zintal, Zinnzallwurzn, Hochwurz, Goldenzian, Bitterwurzn, Meisterwurz, Königswurzn |
| *Gentiana punctata* | 1 | Tupfnwurz |
| *Geranium pratense* | 0 |  |
| *Geranium robertianum* | 5 | Ruprechtskraut, Unser Herrn Nagel, St., Wanzenkräutl, Rotlaufkrutt, Robertkraut |
| *Geranium sanguineum* | 0 |  |
| *Geum montanum* | 2 | Nelkwurz, Bendiktenkräutl |
| *Geum reptans* | 0 |  |
| *Geum rivale* | 0 |  |
| *Geum urbanum* | 0 |  |
| *Glechoma hederacea* | 6 | Gundermann, Donnerrebe, Erdfreu, Wundrebe, Wildes Peterl, Gundelrieme |
| *Globularia cordifolia* | 3 | Blaue Maßlieb, Morgenröschen, Globularie |
| *Hedera helix* | 1 | Eapam |
| *Helianthemum alpestre* | 3 | Sonnenblüml, Goldröslein, Elisabethblüml |
| *Heracleum sphondylium* | 0 |  |
| *Herniaria glabra* | 0 |  |
| *Hieracium intybaceum* | 2 | Altheae, Klebriges-Habichtskraut |
| *Hieracium pilosella* | 0 |  |
| *Hippophae rhamnoides* | 0 |  |
| *Humulus lupulus* | 2 | Kletternder Wolf, Buschhopfen |
| *Hyoscyamus niger* | 2 | Zahnkräutl, Hyosciami |
| *Hypericum maculatum* | 4 | Hartreu, Tüpfelhartreu, Tausenjochkräutl, Blutkraut |
| *Hypericum montanum* | 0 |  |
| *Hypericum perforatum* | 17 | Blutkraut, Elfenblut, Fraunpliester, Harthreu, Herrgotts Wundkraut, Herrgottsblut, Hexenkraut, Jagedenteufel, Jagedeifel, Jungfernzucht, Kuttelkrutt, Liebeskraut, Teufelsfluch, Tüpfelheu, Unser Frauen Bettstroh, Walpurgiskraut, Wundokräutl |
| *Ilex aquifolium* | 1 | Metabaum |
| *Juniperus communis* | 17 | Feuerbaum, Heidewacholder, Krammetsbeere, Kranaweten, Kranaweter, Kranebitt, Kranewit, Kranewittstaude, Kronawötten, Machandl, Räucherstrauch, Reckolder, Stechholder, Wachandl, Wacholder, Wachtelbeerstrauch, Weihrauchbaum |
| *Juniperus communis* var. *saxatilis* | 1 | Kranewitt |
| *Juniperus sabina* | 3 | Sadebaum, Seven, Sebenstrauch |
| *Lamium album* | 0 |  |
| *Lamium galeobdolon* | 3 | Goldnessel, Gelbe Taubnettele |
| *Lamium purpureum* | 0 |  |
| *Larix decidua* | 2 | Lörbaum, Lörgatbaum |
| *Leontopodium nivale* | 7 | Bauchwehblüml, König der Blumen, Alpenruhrkrutt, Irlweis, Strahliges Ruhrkräutl, Liebstern, Samtbluiml |
| *Leonurus cardiaca* | 2 | Herzgespannkräutl, Falscher Andorn |
| *Lepidium sativum* | 1 | Kresse |
| *Leucanthemopsis alpina* | 3 | Orakelbluiml, Flohkrutt, Wucherblüml |
| *Leucanthemum vulgare* | 3 | Orakelbluiml, Flohkrutt, Wucherblüml |
| *Lilium bulbiferum* | 3 | Donnerblüml, Gerg-Ilga, Tulipana |
| *Lilium martagon* | 3 | Berglilie, Goldapfel, Sillichwurzn |
| *Lycopodium clavatum* | 1 | Weidmann |
| *Malva alcea* | 0 |  |
| *Malva neglecta* | 2 | Käsepappel, Rosspappel |
| *Malva sylvestris* | 3 | Käsepappel, Malve, Kaaspapelen |
| *Marrubium vulgare* | 2 | Mariendistel, Helfkräutl |
| *Matricaria chamomilla* | 4 | Kummerbluiml, Muskatbluiml, Gramille, Mutterkräutl |
| *Melilotus officinalis* | 2 | Wiesnrklee, Gelber Steinklee |
| *Mentha aquatica* | 0 |  |
| *Mentha arvensis* | 0 |  |
| *Mentha longifolia* | 2 | Wilde-Minze, Ross-Minze |
| *Mentha pulegium* | 1 | Flohminze |
| *Menyanthes trifoliata* | 2 | Fieberklee, Bitterklee |
| *Mutellina adonidifolia* | 3 | Muttelli, Mattau, Alpenbärenwurzn |
| *Myricaria germanica* | 0 |  |
| *Nasturtium officinale* | 3 | Entgiftungskrutt, Bachkröss, Kröss |
| *Nepeta cataria* | 0 |  |
| *Ononis spinosa* | 0 |  |
| *Origanum vulgare* | 2 | Wilder Mjoran, Dost |
| *Ornithogalum umbellatum* | 0 |  |
| *Papaver rhoeas* | 3 | Feldmohn, Wilder Magen |
| *Parnassia palustris* | 2 | Studentenrösal, Himmlsterndl |
| *Petasites hybridus* | 0 |  |
| *Peucedanum ostruthium* | 9 | Bergwurz, Brustwurzn, Durstwurz, Göttliches Heilmittel, Hirschwurn, Horschtrinse, Kaiserwurz, Moasterwurz, Thomaswurzn |
| *Picea abies* | 3 | Feichte, Rottanne, Schwarztanne |
| *Pimpinella major* | 2 | Bockwurz, Theriakwurzn |
| *Pimpinella saxifraga* | 8 | Bockwurz, Biberklee, Bimbernell, Steinpeterl, Pockwurschze, Theriakwurzn, Pfefferwurzn, Steinbrech |
| *Pinguicula alpina* | 3 | Fettblattl, Buttergras, Heilblattl |
| *Pinus cembra* | 3 | Zirbelkiefer, Zirm, Arve |
| *Pinus mugo* | 3 | Latsche, Legföhr, Krummholz |
| *Pinus sylvestris* | 1 | Föhr |
| *Plantago lanceolata* | 2 | Herr des Weges, Rossrippe, Hermelkraut |
| *Plantago major* | 2 | Lügenblatt, Breitwegerich |
| *Plantago media* | 0 |  |
| *Polygala chamaebuxus* | 4 | Kreuzblüml, Wilder Buchs, Marienvögele, Alpenbuchsl |
| *Polygonatum odoratum* | 0 |  |
| *Polygonum aviculare* | 0 |  |
| *Polypodium vulgare* | 3 | Engelsüß, Süßwurzn, Steinlakritze |
| *Populus tremula* | 1 | Espe |
| *Potentilla anserina* | 4 | Anserina, Gänserich, Krächtskräutl, Krampfkrutt |
| *Potentilla aurea* | 0 |  |
| *Potentilla erecta* | 2 | Edle-Blutwurzn, Tormentill |
| *Potentilla grandiflora* | 0 |  |
| *Potentilla reptans* | 0 |  |
| *Primula auricula* | 3 | Gamsbleaml, Aurikele, Plantenigel |
| *Primula elatior* | 10 | Echtes-Schlüsselbluemel, Himmelsschlüssel, Kraftblüml, Gichtblüml, Märzenblume, Osterblume, St-Peter-Schlüssel, Fastbleaml, Eieräuglein, Allejuliableaml |
| *Primula farinosa* | 3 | Maiblüml, Rosäugli, Frauenäugl |
| *Primula glutinosa* | 3 | Speik, Blauer-Speik, Roter Speik |
| *Primula matthioli* | 4 | Bergsanikel, Dreifaltigkeitsglöcklein, Alpenglöckli, Wundglöcklein |
| *Primula veris* | 10 | Himmelsschlüssel, Kraftblüml, Himmelsschlüssel, Kraftblüml, Gichtblüml, Märzenblume, Osterblume, St-Peter-Schlüssel, fastebleaml, Eieräuglein, Allejuliableaml, Petrusschlüssel |
| *Primula vulgaris* | 2 | Gartenprimel, Erd-Primel |
| *Prunus avium* | 1 | Kerschn |
| *Prunus spinosa* | 2 | Schlehdorn, Schwarzdorna |
| *Pteridium aquilinum* | 0 |  |
| *Pulmonaria officinalis* | 0 |  |
| *Pyrus pyraster* | 1 | Holz-Birne |
| *Quercus petraea* | 3 | Eich, Oachl, Aichen |
| *Quercus pubescens* | 3 | Aichen, Eich, Oachl |
| *Quercus robur* | 2 | Aichen, Oachl, Eich |
| *Ranunculus acris* | 1 | Schmerzpfandel |
| *Beckwithia glacialis* | 3 | Gletscherrösal, Gamskreß, Rükkehrzu |
| *Raphanus raphanistrum* | 3 | Hederich, Radi, Winterrettich |
| *Rhamnus frangula* | 1 | Grindholz |
| *Sedum roseum* | 2 | Frauenzopf, Frauenwurzn |
| *Rhododendron ferrugineum* | 3 | Oswaldstaude, Almrausch, Rosenholz |
| *Ribes petraeum* | 2 | Zaufen, Ribisl |
| *Rosa canina* | 4 | Heckenroasn, Hundsrose, Dornäpfel, Dornapfelen |
| *Rosa corymbifera* | 0 |  |
| *Rosa montana* | 1 | Roasn |
| *Rosa pendulina* | 3 | Berhgsrosan, Dirndalrosan, Mairose |
| *Rubus idaeus* | 1 | Kreinbeer |
| *Rubus bifrons* | 2 | Kratzbeer, Schwarzbeer |
| *Rumex acetosa* | 0 |  |
| *Rumex alpinus* | 0 |  |
| *Rumex crispus* | 0 |  |
| *Rumex scutatus* | 0 |  |
| *Ruscus aculeatus* | 0 |  |
| *Salix alba* | 3 | Felba, Feler, Felerwind |
| *Salix caprea* | 3 | Salch, Hoft, Mitzeleholz |
| *Salix pentandra* | 0 |  |
| *Salix purpurea* | 0 |  |
| *Salix serpyllifolia* | 1 | Quendelweide |
| *Salvia glutinosa* | 2 | Leimsalbei, Bergsalbei |
| *Sambucus ebulus* | 3 | Attich, Miete, Adach |
| *Sambucus nigra* | 3 | Holler, Holder, Schwarzer Flieder, Mausflieder |
| *Sambucus racemosa* | 3 | Holler, Holder, Berghollunder |
| *Sanguisorba minor* | 0 |  |
| *Sanicula europaea* | 4 | Niklaswurzen, Heil aller Schäden, Wald Sanikel, Heildolde |
| *Saponaria officinalis* | 1 | Seifenkräutl |
| *Scrophularia nodosa* | 0 |  |
| *Sedum atratum* | 0 |  |
| *Sedum telephium* | 0 |  |
| *Sempervivum montanum* | 3 | Donnerkraut, Feuerkräutl, Wetterwurz |
| *Sempervivum tectorum* | 4 | Dachwurz, Donnerkraut, Hauslaub, Steinäpfel |
| *Silene acaulis* | 3 | Polster-Leimkraut, Teufelspeitsche, Niesnagerl |
| *Silene vulgaris* | 1 | Schnellerkraut |
| *Solanum dulcamara* | 3 | Alpranke, Mäseholz, Heischkraut |
| *Soldanella alpina* | 3 | Beschreikräutl, Eisglöckchen, Bärgglöggeli |
| *Solidago virgaurea* | 3 | Fuchsschwanz, Goldene Jungfrau, Heidnisch Wundkräutl |
| *Sorbus aucuparia* | 3 | Vogelbeer, Mostber, Faulischbeer |
| *Stachys sylvatica* | 3 | Bienensaug, Stinkblatt, Klaffen |
| *Stellaria media* | 4 | Sternmiere, Vogelmiere, Hühnerdarm, Hühnergras |
| *Symphytum officinale* | 3 | Schwarzwurz, Himmlbrot, Wallwurz |
| *Tanacetum vulgare* | 0 |  |
| *Taraxacum campylodes* | 7 | Kuhbluiml, Zigori, Butterblüml, Augemilchkräutl, Bettseicher, Milchbluiml, Pfaffenröhrlein |
| *Thlaspi rotundifolium* | 0 |  |
| *Thymus pulegioides* | 4 | Quendel, Wilder Thymian, Karwendel, Kinderbadkräutl |
| *Tilia cordata* | 4 | Stein-, Berg-, Spät-und Waldlinde |
| *Tilia platyphyllos* | 2 | Bastholz, Lindt |
| *Tragopogon dubius* | 1 | Bochsbatschlan |
| *Trifolium pratense* | 4 | Rotklee, Himmelbrot, Zuckerblüml, Honigblume |
| *Trifolium repens* | 1 | Kriech-Klee |
| *Tussilago farfara* | 3 | Eselstritt, Märzbluma, Bachblümlein, Hustenblümli |
| *Urtica dioica* | 5 | Nettelle, Gichtruta, Haarnessel, Feuerkräutl, Nesselmännlein |
| *Urtica urens* | 1 | Nessel |
| *Usnea barbata* | 2 | Baummoos, Altmännerbart |
| *Usnea dasopoga* | 1 | Baumbart |
| *Vaccinium myrtillus* | 4 | Schwarzbeer, Swotschba, Blaubeer, Moschbeer |
| *Vaccinium vitis-idaea* | 3 | Granten, Rauschgranate, Kreuzbeer |
| *Valeriana montana* | 1 | Speik |
| *Valeriana officinalis* | 10 | Aldrianwurzen, Augenwurz, Äugleinkräutl, Ballerjan, Elfenpflanze, Hexenkraut, Katzenkraut, Katzenwurz, Mondwurz, Speik |
| *Veratrum album* | 3 | Hammanwurzel, Laiskraut, Schemel |
| *Verbascum densiflorum* | 5 | Himmelbrand, Wollkraut, Fackelkrätl, Marienkerze, Wundblüml |
| *Verbascum phlomoides* | 3 | Wollkraut, Wetterkerze, Himmelbrand |
| *Verbascum thapsus* | 2 | Himmelbrand, Wollkraut |
| *Verbena officinalis* | 3 | Venusader, Springwurz, Träner der Isis |
| *Veronica alpina* | 1 | Männertreu, Grindheil |
| *Veronica chamaedrys* | 9 | Gamander, Männertreu, Sylvesterbluiml, Unser Lieben Frau Rast, Blüht der Gipfel nicht, blüht doch da und dort ein Ast, Beisswurmkräutl, Wundkrutt, Viehkräutl, Grindheil |
| *Veronica officinalis* | 3 | Wald-Ehrenpreis, Männertrau, Grindheil |
| *Viola biflora* | 3 | Gelbvigerl, Gelveilchen |
| *Viola odorata* | 1 | Dreifaltigkeitsbluimal |
| *Viola tricolor* | 4 | Stiefmütterchen, Schwiegerblümlein, Ackerveilchen, Dreifaltigkeitsbluimal |
| *Viscum album* | 4 | Trudenfuß, Wintergrün, Muschgl, Kruschgl |
