# Supplementary material for: Traditional medicinal plants in South Tyrol (northern Italy, southern Alps): biodiversity and use
Source: J Ethnobiol Ethnomed. 2020 Nov 26;16:74. doi: 10.1186/s13002-020-00419-8 (PMC7690129; doi:10.1186/s13002-020-00419-8)
Supplement: Supplementary file 3 — Additional file 3. Appendix C. [file 13002_2020_419_MOESM3_ESM.docx]

Appendix C

Native plants used in traditional folk medicine in the region of South Tyrol (Alto Adige, Italy)

| Nr. | Plant Species | Family | Growth form | Citation frequency | Vernacular names | Plant parts used | Use versatility | Medicinal uses^1^ | Pharmacological or Phytochemical evidence | Protection status (Protected; partially protected; not protected) | Red List Status | Wild or Cultivated | Not used anymore |
| --- | --- | --- | --- | --- | --- | --- | --- | --- | --- | --- | --- | --- | --- |
| 1 | *Abies alba* Mill. | Pinaceae | Tree | 4 | 1 | Leaf | 3 | [4]; [5]; [7]; [9]; [10] | n.a. | Partially protected | LC | Cultivated |  |
| 2 | *Achillea atrata* L. | Asteraceae | Herb | 2 | 3 | Bud; Flower; Leaf | 4 | [5]; [6]; [7]; [8]; [9]; [11]; [12] | n.a. | Not protected | LC | Wild |  |
| 3 | *Achillea clavennae* L. | Asteraceae | Herb | 1 | 4 | Flower; Leaf | 4 | [5]; [6]; [7]; [8]; [9]; [11]; [12] | n.a. | Not protected | LC | Wild |  |
| 4 | *Achillea millefolium* L. | Asteraceae | Herb | 12 | 17 | Bud; Flower; Leaf | 4 | [2]; [6]; [7], [8]; [10]; [11]; [12) | Positive | Not protected | LC | Cultivated |  |
| 5 | *Achillea moschata* Jacq. | Asteraceae | Herb | 10 | 4 | Flower; Leaf | 4 | [1]; [6]; [7]; [8]; [9]; [11]; [12] | n.a. | Not protected | LC | Wild |  |
| 6 | *Aconitum napellus* L. | Ranunculaceae | Herb | 4 | 3 | Leaf; Root | 2 | [1] | Negative | Not protected | LC | Cultivated |  |
| 7 | *Adiantum capillus-veneris* L. | Adiantaceae | Fern | 2 | 4 | Leaf | 1 | [8]; [12] | n.a. | Protected | VU | Wild | x |
| 8 | *Aegopodium podagraria L*. | Apiaceae | Herb | 5 | 6 | Leaf; Root | 2 | [1]; [8]; [9]; [10] | n.a. | Not protected | LC | Wild |  |
| 9 | *Aesculus hippocastanum* L. | Hippocastanaceae | Tree | 5 | 1 | Flower; Fruit; Leaf | 2 | [1]; [9]; [10] | Positive | Not protected | NE | Wild |  |
| 10 | *Agrimonia eupatoria* auct. | Rosaceae | Herb | 5 | 2 | Flower; Leaf | 1 | [7]; [8]; [9]; [11] | Positive | Not protected | LC | Cultivated |  |
| 11 | *Ajuga reptans* L. | Lamiaceae | Herb | 1 | 0 | Flower; Leaf | 1 | [10] | n.a. | Not protected | LC | Wild |  |
| 12 | *Alchemilla alpina* L. | Asteraceae | Herb | 9 | 3 | Flower; Leaf; Root | 4 | [1]; [2]; [3]; [6]; [8]; [9]; [11]; [12] | Negative | Not protected | DD | Wild |  |
| 13 | *Alchemilla xanthochlora* Rothm. | Rosaceae | Herb | 12 | 18 | Flower; Leaf; Root | 5 | [1]; [3]; [7]; [8]; [9]; [10]; [12] | Positive | Not protected | LC | Cultivated |  |
| 14 | *Alliaria petiolate* (M.Bieb.) Cavara & Grande | Brassicaceae | Herb | 2 | 0 | Leaf | 2 | [1]; [3]; [6]; [8]; [11] | n.a. | Not protected | LC | Wild |  |
| 15 | *Allium schoenoprasum* L. | Liliaceae | Herb | 4 | 2 | Leaf | 4 | [1]; [4]; [5]; [6]; [7]; [9]; [10]; [11] | n.a. | Not protected | LC | Cultivated |  |
| 16 | *Allium ursinum* L. | Liliaceae | Herb | 10 | 5 | Bud; Flower; Leaf | 2 | [3]; [6]; [7]; [8]; [9] | n.a. | Not protected | VU | Wild |  |
| 17 | *Allium victorialis* L. | Amaryllidaceae | Herb | 2 | 2 | Leaf; Root | 2 | [1]; [6]; [8]; [11] | n.a. | Not protected | LC | Wild |  |
| 18 | *Althaea officinalis* L. | Malvaceae | Herb | 11 | 4 | Flower; Leaf; Root | 3 | [1]; [4]; [7]; [8]; [9]; [10] | Positive | Not protected | NT | Cultivated |  |
| 19 | *Anacamptis morio* (L.) R.M.Bateman, Pridgeon & M.W.Chase | Orchidaceae | Herb | 1 | 0 | Root | 1 | [9] | n.a. | Protected | VU | Wild |  |
| 20 | *Anemone vernalis* L. | Ranunculaceae | Herb | 1 | 2 | Flower; Leaf | 1 | [8]; [9]; [10]; [11] | Negative | Protected | LC | Wild |  |
| 21 | *Angelica sylvestris* L. | Apiaceae | Herb | 10 | 0 | Flower; Leaf; Root; Seed | 3 | [4]; [6]; [7]; [8]; [10] | Positive | Not protected | LC | Wild |  |
| 22 | *Antennaria dioica* (L.) Gaertn. | Asteraceae | Herb | 1 | 3 | Flower | 2 | [7]; [11] | Negative | Not protected | LC | Wild | x |
| 23 | *Anthriscus sylvestris* (L.) Hoffm. | Apiaceae | Herb | 2 | 1 | Leaf; Root | 2 | [5]; [8]; [10]; [11] | n.a. | Not protected | LC | Wild |  |
| 24 | *Anthyllis vulneraria* L. | Fabaceae | Herb | 1 | 3 | Flower; Leaf | 1 | [1]; [9] | n.a. | Not protected | LC | Wild |  |
| 25 | *Aquilegia einseleana* F.W.Schultz | Ranunculaceae | Herb | 2 | 0 | Flower | 1 | [7] | n.a. | Protected | LC | Cultivated |  |
| 26 | *Arabis petiolata* M. Bieb. | Brassicaceae | Herb | 1 | 0 | Fruit; Leaf; Seed | 2 | [1]; [9] | n.a. | Not protected | LC | Wild |  |
| 27 | *Arctium lappa* L. | Asteraceae | Herb | 5 | 1 | Leaf; Root | 4 | [8]; [9]; [10]; [11] | Negative | Not protected | LC | Wild |  |
| 28 | *Arctostaphylos uva-ursi* (L.) Spreng. | Ericaceae | Shrub | 3 | 7 | Leaf | 1 | [8]; [11] | Positive | Not protected | LC | Wild |  |
| 29 | *Arnica montana* L. | Asteraceae | Herb | 13 | 18 | Flower; Root | 4 | [1]; [4]; [6] [7]; [8]; [9]; [10]; [12] | Positive | Partially protected | LC | Cultivated |  |
| 30 | *Artemisia absinthium* L. | Asteraceae | Herb | 10 | 9 | Flower; Leaf | 5 | [1]; [3]; [6] [7]; [8]; [11]; [12] | Positive | Not protected | LC | Cultivated |  |
| 31 | *Artemisia mutellina* Vill. | Asteraceae | Herb | 4 | 3 | Leaf | 3 | [1]; [4]; [7]; [8]; [11]; [12] | n.a. | Not protected | LC | Wild |  |
| 32 | *Artemisia vulgaris* L | Asteraceae | Herb | 9 | 5 | Leaf; Root; Seed | 4 | [1]; [4]; [6] [7]; [8]; [11]; [12] | Negative | Not protected | LC | Cultivated |  |
| 33 | *Asparagus officinalis* L. | Liliaceae | Herb | 1 | 0 | Root | 2 | [8] | Negative | Not protected | LC | Cultivated |  |
| 34 | *Asplenium septentrionale* (L.) Hoffm. | Aspleniaceae | Fern | 1 | 3 | Leaf | 1 | [10] | n.a. | Not protected | LC | Wild |  |
| 35 | *Athamanta cretensis* L. | Apiaceae | Herb | 1 | 3 | Leaf; Root; Seed | 2 | [8]; [11]; [12] | n.a. | Not protected | LC | Wild |  |
| 36 | *Atropa belladonna* L. | Solanaceae | Herb | 2 | 0 | Root | 1 | [4]; [10] | Positive | Not protected | LC | Cultivated |  |
| 37 | *Auricularia auricula-judae* (Bull.) Wettst. | Auriculariaceae | Mushroom | 3 | 2 | Fruit | 2 | [5]; [6]; [7]; [10] | n.a. | Not protected | NE | Wild |  |
| 38 | *Bellis perennis* L. | Asteraceae | Herb | 7 | 6 | Flower | 3 | [6]; [7]; [9] | n.a. | Not protected | LC | Cultivated |  |
| 39 | *Berberis vulgaris* L. | Berberidaceae | Shrub | 7 | 4 | Bark; Fruit | 3 | [6]; [7]; [8]; [9]; [11] | Negative | Not protected | LC | Cultivated |  |
| 40 | *Betonica officinalis* L. | Lamiaceae | Herb | 1 | 2 | Flower; Leaf | 1 | [4]; [7]; [8] | n.a. | Not protected | LC | Wild |  |
| 41 | *Betula pendula* Roth | Betulaceae | Tree | 11 | 4 | Barl; Leaf; Resin | 5 | [1]; [6]; [8]; [9]; [10]; [11] | Positive | Not protected | LC | Wild |  |
| 42 | *Biscutella laevigata* L. | Brassicaceae | Herb | 1 | 1 | Flower; Leaf | 1 | [1]; [9]; [11] | n.a. | Not protected | LC | Wild | x |
| 43 | *Borago officinalis* L. | Boraginaceae | Herb | 3 | 0 | Flower; Leaf | 2 | [7]; [10]; [11] | Negative | Not protected | NE | Cultivated |  |
| 44 | *Botrychium lunaria* (L.) Sw. | Ophioglossaceae | Fern | 2 | 3 | Leaf; Root | 2 | [12] | n.a. | Protected | LC | Wild | x |
| 45 | *Brassica rapa* L. | Brassicaceae | Herb | 3 | 2 | Leaf; Root | 3 | [1]; [2]; [7]; [8]; [11] | n.a. | Not protected | LC | Cultivated |  |
| 46 | *Calluna vulgaris* (L.) Hull | Ericaceae | Shrub | 4 | 4 | Flower;Leaf | 2 | [4]; [6]; [8]; [10]; [11] | Negative | Not protected | LC | Wild |  |
| 47 | *Campanula patula* L. | Campanulaceae | Herb | 1 | 2 | Flower;Leaf | 1 | [5]; [4]; [7] | n.a. | Not protected | LC | Wild |  |
| 48 | *Campanula rotundifolia* L. | Campanulaceae | Herb | 2 | 2 | Flower;Leaf | 1 | [5]; [4]; [7] | n.a. | Not protected | LC | Cultivated |  |
| 49 | *Capsella bursa-pastoris* (L.) Medik | Brassicaceae | Herb | 9 | 4 | Flower; Fruit; Leaf | 2 | [6]; [8]; [9]; [10]; [11]; [12] | Positive | Not protected | LC | Wild |  |
| 50 | *Carlina acaulis* L. | Asteraceae | Herb | 3 | 8 | Flower | 4 | [7]; [8] | n.a. | Not protected | LC | Wild |  |
| 51 | *Carum carvi* L. | Apiaceae | Herb | 8 | 4 | Flower; Leaf; Seed | 2 | [6]; [8]; [10]; [11] | Positive | Not protected | LC | Cultivated |  |
| 52 | *Castanea sativa* Mill. | Fagaceae | Tree | 2 | 2 | Fruit; Leaf | 4 | [6]; [7] | Negative | Not protected | LC | Wild |  |
| 53 | *Centaurium erythraea* Rafn | Gentianaceae | Herb | 5 | 3 | Flower; Leaf | 2 | [2]; [6]; [8]; [9]; [11] | Positive | Protected | NT | Wild |  |
| 54 | *Cerastium fontanum* Baumg. | Caryophyllaceae | Herb | 1 | 0 | Flower; Leaf | 1 | [5] | n.a. | Not protected | LC | Wild |  |
| 55 | *Cetraria islandica* (L.) Ach. | Parmeliaceae | Moss | 8 | 12 | Leaf | 3 | [2]; [4]; [6]; [7]; [8]; [9] | Positive | Not protected | NE | Wild |  |
| 56 | *Chelidonium majus* L. | Papaveraceae | Herb | 8 | 3 | Leaf; Resin; Root | 2 | [5]; [8]; [9]; [10] | Positive | Not protected | LC | Cultivated |  |
| 57 | *Chenopodium bonus-henricus* L. | Chenopodiaceae | Herb | 3 | 2 | Flower; Fruit; Leaf; Root | 2 | [1]; [6]; [8]; [9] | n.a. | Not protected | LC | Wild |  |
| 58 | *Cichorium intybus* L. | Asteraceae | Herb | 5 | 3 | Flower;Leaf; Root | 3 | [1]; [4]; [5]; [7]; [8]; [9]; [10] | Positive | Not protected | LC | Cultivated |  |
| 59 | *Clinopodium alpinum* (L.) Kuntze | Lamiaceae | Herb | 2 | 3 | Flower;Leaf | 2 | [4]; [8] | n.a. | Not protected | LC | Cultivated |  |
| 60 | *Corylus avellana* L. | Betulaceae | Shrub | 3 | 2 | Bark; Fruit; Leaf | 5 | [4]; [6] [8] | n.a. | Not protected | LC | Wild |  |
| 61 | *Crataegus monogyna* Jacq. | Rosaceae | Shrub | 11 | 7 | Flower; Fruit; Leaf | 3 | [3]; [4]; [6]; [7] | Positive | Not protected | LC | Wild |  |
| 62 | *Cyanus segetum* Hill | Asteraceae | Herb | 1 | 3 | Flower | 2 | [8]; [9]; [11] | Negative | Not protected | EN | Wild |  |
| 63 | *Cyclamen purpurascens* Mill. | Primulaceae | Herb | 1 | 3 | Root | 2 | [9]; [12] | n.a. | Protected | LC | Wild |  |
| 64 | *Daucus carota* L. | Apiaceae | Herb | 3 | 2 | Root | 2 | [5]; [6]; [8]; [12] | n.a. | Not protected | LC | Wild |  |
| 65 | *Dentaria enneaphyllos* L. | Brassicaceae | Herb | 1 | 3 | Root | 2 | [10] | n.a. | Not protected | LC | Wild |  |
| 66 | *Diplotaxis tenuifolia* (L.) DC. | Brassicaceae | Herb | 1 | 1 | Leaf | 2 | [1] | n.a. | Not protected | LC | Wild |  |
| 67 | *Dipsacus fullonum* L. | Caprifoliaceae | Herb | 1 | 0 | Root | 1 | [1]; [10] | n.a. | Not protected | EN | Wild |  |
| 68 | *Drosera rotundifolia* L. | Droseraceae | Herb | 1 | 0 | Leaf | 1 | [2]; [9] | Positive | Protected | NT | Wild |  |
| 69 | *Dryas octopetala* L. | Rosaceae | Herb | 3 | 4 | Flower; Leaf | 2 | [1]; [4]; [6]; [7] | n.a. | Not protected | LC | Wild |  |
| 70 | *Dryopteris filix-mas* (L.) Schott | Dryopteridaceae | Fern | 5 | 10 | Leaf; Root | 3 | [1]; [8]; [9]; [10] | Negative | Not protected | LC | Wild |  |
| 71 | *Elymus repens* (L.) Gould | Poaceae | Herb | 4 | 1 | Leaf; Root | 1 | [6]; [7] | Positive | Not protected | LC | Wild |  |
| 72 | *Epilobium angustifolium* L. | Onagraceae | Herb | 4 | 1 | Flower | 1 | [11] | n.a. | Not protected | LC | Wild |  |
| 73 | *Epilobium montanum* L. | Onagraceae | Herb | 2 | 0 | Flower | 1 | [11] | n.a. | Not protected | LC | Wild |  |
| 74 | *Epilobium palustre* L. | Onagraceae | Herb | 1 | 0 | Flower | 1 | [11] | n.a. | Not protected | LC | Wild |  |
| 75 | *Epilobium parviflorum* Schreb. | Onagraceae | Herb | 7 | 1 | Flower; Leaf | 1 | [11] | n.a. | Not protected | LC | Wild |  |
| 76 | *Equisetum arvense* L. | Equisetaceae | Fern | 13 | 4 | Leaf | 5 | [1]; [3]; [4]; [5]; [6]; [7]; [8]; [9]; [10]; [11; [12] | Positive | Not protected | LC | Wild |  |
| 77 | *Equisetum palustre* L. | Equisetaceae | Fern | 1 | 1 | Leaf | 1 | [9] | n.a. | Not protected | LC | Wild |  |
| 78 | *Equisetum pratense* Ehrh. | Equisetaceae | Fern | 3 | 3 | Leaf | 2 | [9] | n.a. | Not protected | LC | Wild |  |
| 79 | *Erica carnea* L. | Ericaceae | Shrub | 1 | 1 | Flower; Leaf | 2 | [6]; [8]; [11] | n.a. | Not protected | LC | Wild |  |
| 80 | *Erigeron alpinus* L. | Asteraceae | Herb | 1 | 3 | Leaf | 2 | [1]; [9]; [12] | n.a. | Not protected | LC | Wild |  |
| 81 | *Eriophorum angustifolium* Honck. | Cyperaceae | Herb | 2 | 2 | Flower | 1 | [1]; [9] | n.a. | Not protected | LC | Wild |  |
| 82 | *Eryngium amethystinum* L. | Apiaceae | Herb | 1 | 0 | Flower | 1 | [8] | n.a. | Not protected | EX | Wild |  |
| 83 | *Eryngium campestre* L. | Apiaceae | Herb | 1 | 0 | Flower | 1 | [8]; [7] | n.a. | Not protected | EX | Wild |  |
| 84 | *Euphrasia alpina* (L.) Bubani | Orobanchaceae | Herb | 1 | 0 | Flower; Leaf | 1 | [5]; [8] | n.a. | Not protected | LC | Wild |  |
| 85 | *Euphrasia minima* Jacq. | Orobanchaceae | Herb | 1 | 0 | Flower; Leaf | 1 | [5]; [8] | n.a. | Not protected | LC | Wild |  |
| 86 | *Euphrasia officinalis* L. | Orobanchaceae | Herb | 9 | 10 | Flower; Leaf | 2 | [5]; [8] | Negative | Not protected | LC | Wild | x |
| 87 | *Euphrasia rostkoviana* Hayne | Orobanchaceae | Herb | 6 | 3 | Flower; Leaf | 1 | [5]; [4]; [7]; [8] | n.a. | Not protected | LC | Wild |  |
| 88 | *Euphrasia stricta* D. Wolff | Orobanchaceae | Herb | 1 | 0 | Flower; Leaf | 1 | [5]; [8] | n.a. | Not protected | LC | Wild |  |
| 89 | *Euphrasia versicolor* A. Kern. | Orobanchaceae | Herb | 1 | 0 | Flower; Leaf | 1 | [5]; [8] | n.a. | Not protected | LC | Wild |  |
| 90 | *Fagus sylvatica* L. | Fagaceae | Tree | 2 | 0 | Bark; Leaf | 4 | [1]; [7]; [9] | n.a. | Not protected | LC | Wild |  |
| 91 | *Filipendula ulmaria* (L.) Maxim. | Rosaceae | Herb | 5 | 5 | Flower; Leaf | 3 | [1]; [7]; [8]; [9]; [10] | Positive | Not protected | LC | Wild |  |
| 92 | *Fomitopsis betulina (Bull*.*) B*.*K*.*Cui*, *M*.*L*.*Han & Y*.*C*.*Dai* | Fomitopsidaceae | Mushroom | 1 | 0 | Fruit |  | [1] | n.a. | Not protected | NE | Wild |  |
| 93 | *Fomitopsis officinalis* (Vill.) Bondartsev & Singer | Fomitopsidaceae | Mushroom | 1 | 0 | Fruit | 1 | [6] | n.a. | Not protected | NE | Wild | x |
| 94 | *Fragaria vesca* L. | Rosaceae | Herb | 5 | 1 | Fruit; Leaf | 3 | [1]; [2]; [6]; [9]; [10] | Negative | Not protected | LC | Wild |  |
| 95 | *Fragaria viridis* Weston | Rosaceae | Herb | 1 | 1 | Fruit; Leaf | 2 | [1]; [2]; [6]; [9]; [10] | n.a. | Not protected | LC | Wild |  |
| 96 | *Fraxinus excelsior* L. | Oleaceae | Tree | 3 | 2 | Bark; Bud; Fruit; Leaf | 3 | [5]; [8]; [9]; [10]; [11] | Negative | Not protected | LC | Wild |  |
| 97 | *Fraxinus ornus* L. | Oleaceae | Tree | 1 | 2 | Flower; Leaf; resin | 1 | [4]; [8]; [10] | n.a. | Not protected | LC | Wild |  |
| 98 | *Fumaria officinalis* L. | Papaveraceae | Herb | 6 | 3 | Leaf | 1 | [6]; [8]; [9] | Positive | Not protected | LC | Wild |  |
| 99 | *Galega officinalis* L. | Fabaceae | Herb | 1 | 2 | Flower; Leaf | 1 | [2]; [11] | Negative | Not protected | NT | Wild |  |
| 100 | *Galeopsis pubescens* Besser | Lamiaceae | Herb | 1 | 0 | Leaf | 1 | [1]; [7]; [8]; [9] | n.a. | Not protected | LC | Wild |  |
| 101 | *Galium aparine* L. | Rubiaceae | Herb | 2 | 0 | Flower; Leaf | 2 | [8]; [9] | n.a. | Not protected | LC | Wild |  |
| 102 | *Galium mollugo* L. | Rubiaceae | Herb | 3 | 2 | Leaf | 3 | [2]; [7]; [9]; | n.a. | Not protected | LC | Wild |  |
| 103 | *Galium odoratum* (L.) Scop. | Rubiaceae | Herb | 4 | 2 | Leaf | 3 | [2]; [3]; [4]; [6] | Negative | Not protected | LC | Wild |  |
| 104 | *Galium verum* L. | Rubiaceae | Herb | 5 | 2 | Leaf | 2 | [8]; [9]; [11] | n.a. | Not protected | LC | Wild | x |
| 105 | *Genista tinctoria* L. | Fabaceae | Shrub | 1 | 0 | Flower;Leaf | 1 | [11] | n.a. | Not protected | LC | Wild |  |
| 106 | *Gentiana acaulis* L. | Gentianaceae | Herb | 1 | 3 | Root | 2 | [3]; [4]; [5] | n.a. | Protected | LC | Wild |  |
| 107 | *Gentiana lutea* L. | Gentianaceae | Herb | 11 | 7 | Root | 3 | [1]; [5]; [6]; [7]; [8]; [10] | Positive | Partially protected | VU | Wild |  |
| 108 | *Gentiana punctata* L. | Gentianaceae | Herb | 2 | 1 | Root | 1 | [8]; [10] | n.a. | Protected | LC | Wild |  |
| 109 | *Geranium pratense* L. | Geraniaceae | Herb | 1 | 0 | Leaf | 1 | [1]; [4]; [7]; [8]; [9] | n.a. | Not protected | LC | Wild |  |
| 110 | *Geranium robertianum* L. | Geraniaceae | Herb | 8 | 5 | Leaf | 2 | [1]; [4]; [7]; [9]; [10]; [11] | n.a. | Not protected | LC | Wild |  |
| 111 | *Geranium sanguineum* L. | Geraniaceae | Herb | 1 | 0 | Flower | 2 | [4]; [6], [8] | n.a. | Not protected | LC | Wild |  |
| 112 | *Geum montanum* L. | Rosaceae | Herb | 4 | 2 | Flower;Leaf; Root | 1 | [4]; [8]; [11] | n.a. | Not protected | LC | Wild |  |
| 113 | *Geum reptans* L. | Rosaceae | Herb | 1 | 0 | Flower;Leaf; Root | 1 | [4]; [6], [8] | n.a. | Not protected | LC | Wild |  |
| 114 | *Geum rivale* L. | Rosaceae | Herb | 1 | 0 | Flower;Leaf; Root | 1 | [4]; [6], [8] | n.a. | Not protected | LC | Wild |  |
| 115 | *Geum urbanum* L. | Rosaceae | Herb | 2 | 0 | Flower;Leaf; Root | 2 | [4]; [6], [8] | n.a. | Not protected | LC | Wild |  |
| 116 | *Glechoma hederacea* L. | Lamiaceae | Herb | 8 | 6 | Flower;Leaf | 2 | [4]; [7], [8]; [10]; [12] | Positive | Not protected | LC | Wild |  |
| 117 | *Globularia cordifolia* L. | Plantaginaceae | Herb | 1 | 3 | Leaf | 1 | [8]; [12] | n.a. | Not protected | LC | Wild |  |
| 118 | *Hedera helix* L. | Araliaceae | Shrub | 4 | 1 | Leaf | 2 | [7]; [9] | Positive | Not protected | LC | Cultivated |  |
| 119 | *Helianthemum alpestre* (Jacq.) DC. | Cistaceae | Herb | 1 | 3 | Flower;Leaf | 1 | [1]; [4], [9] | n.a. | Not protected | LC | Wild |  |
| 120 | *Heracleum sphondylium* L. | Apiaceae | Herb | 1 | 0 | Leaf; Root | 2 | [11] | n.a. | Not protected | NE | Wild |  |
| 121 | *Herniaria glabra* L. | Caryophyllaceae | Herb | 1 | 0 | Leaf | 1 | [7], [8]; [10]; [11] | Negative | Not protected | LC | Wild |  |
| 122 | *Hieracium intybaceum* Lam. | Asteraceae | Herb | 1 | 2 | Flower | 1 | [1]; [9] | n.a. | Not protected | LC | Wild |  |
| 123 | *Hieracium pilosella* L. | Asteraceae | Herb | 3 | 0 | Flower;Leaf | 1 | [1], [4]; [5]; [8]; [9] | n.a. | Not protected | LC | Wild |  |
| 124 | *Hippophae rhamnoides* L. | Elaeagnaceae | Shrub | 4 | 0 | Fruit | 3 | [1], [3]; [6]; [8]; [11] | n.a. | Not protected | LC | Wild |  |
| 125 | *Humulus lupulus* L. | Cannabaceae | Shrub | 7 | 2 | Fruit | 3 | [3]; [4]; [8]; [9] | Positive | Not protected | LC | Wild |  |
| 126 | *Hyoscyamus niger* L. | Solanaceae | Herb | 3 | 2 | Flower;Leaf | 2 | [8]; [10] | n.a. | Not protected | VU | Cultivated |  |
| 127 | *Hypericum maculatum* Crantz | Hypericaceae | Herb | 2 | 4 | Flower;Leaf | 1 | [1], [3]; [4]; [8]; [9] | n.a. | Not protected | LC | Wild |  |
| 128 | *Hypericum montanum* L. | Hypericaceae | Herb | 1 | 0 | Flower;Leaf | 1 | [4]; [10] | n.a. | Not protected | LC | Wild |  |
| 129 | *Hypericum perforatum* L. | Hypericaceae | Herb | 15 | 17 | Flower;Leaf | 5 | [1], [3]; [4]; [8]; [9]; [10]; [12] | Positive | Not protected | LC | Cultivated |  |
| 130 | *Ilex aquifolium* L. | Aquifoliaceae | Shrub | 1 | 1 | Leaf | 1 | [6] | n.a. | Protected | VU | Wild |  |
| 131 | *Juniperus communis* L. | Cupressaceae | Tree | 13 | 17 | Fruit, Leaf | 4 | [1], [6]; [7]; [8]; [9]; [10]; [11]; [12] | Positive | Not protected | LC | Cultivated |  |
| 132 | *Juniperus communis* var. *saxatilis* Pall. | Cupressaceae | Shrub | 1 | 1 | Fruit, Leaf | 2 | [1], [7]; [8]; [9] | n.a. | Not protected | LC | Wild |  |
| 133 | *Juniperus sabina* L. | Cupressaceae | Shrub | 4 | 3 | Leaf | 2 | [1], [7]; [8]; | n.a. | Not protected | LC | Cultivated |  |
| 134 | *Lamium album* L. | Lamiaceae | Herb | 6 | 0 | Flower,Leaf; Root | 2 | [1], [6]; [7] [8]; [9]; [10]; [11]; [12] | Positive | Not protected | LC | Cultivated |  |
| 135 | *Lamium galeobdolon* subsp. *flavidum* (F.Herm.) Á.Löve & D.Löve | Lamiaceae | Herb | 1 | 3 | Flower;Leaf | 1 | [11] | n.a. | Not protected | NE | Wild |  |
| 136 | *Lamium purpureum* L. | Lamiaceae | Herb | 1 | 0 | Flower; Root | 2 | [1], [5]; [6]; [7] [8]; [10]; [11]; [12] | n.a. | Not protected | LC | Wild |  |
| 137 | *Larix decidua* Mill. | Pinaceae | Tree | 7 | 2 | Bark; Bud; Leaf; Resin | 4 | [1]; [6]; [7] [9]; [10]; [11] | Positive | Not protected | LC | Wild |  |
| 138 | *Leontopodium nivale* (Ten.) Huet ex Hand.-Mazz. | Asteraceae | Herb | 7 | 7 | Flower,Leaf; Root | 4 | [1]; [8]; [9] | n.a. | Not protected | LC | Wild |  |
| 139 | *Leonurus cardiaca* L. | Lamiaceae | Herb | 4 | 2 | Flower,Leaf | 1 | [4] [6]; [10]; [11] | Positive | Not protected | NT | Wild |  |
| 140 | *Lepidium sativum* L. | Brassicaceae | Herb | 1 | 1 | Leaf | 3 | [1]; [2]; [7]; [8]; [9] | n.a. | Not protected | NE | Cultivated |  |
| 141 | *Leucanthemopsis alpina* (L.) Heywood | Asteraceae | Herb | 3 | 3 | Flower | 4 | [1]; [4]; [11] | n.a. | Not protected | LC | Wild |  |
| 142 | *Leucanthemum vulgare* (Vaill.) Lam. | Asteraceae | Herb | 1 | 3 | Flower | 4 | [1]; [4]; [11] | n.a. | Not protected | LC | Wild |  |
| 143 | *Lilium bulbiferum* L. | Liliaceae | Herb | 3 | 3 | Flower; Root | 5 | [1]; [7]; [9]; [12] | n.a. | Protected | NT | Cultivated |  |
| 144 | *Lilium martagon* L. | Liliaceae | Herb | 3 | 3 | Flower; Root | 2 | [1]; [4]; [7]; [9] | n.a. | Protected | LC | Cultivated |  |
| 145 | *Lycopodium clavatum* L. | Lycopodiaceae | Herb | 2 | 1 | Leaf; Seed | 1 | [4]; [8]; [10] | n.a. | Partially protected | LC | Wild |  |
| 146 | *Malva alcea* L. | Malvaceae | Herb | 1 | 0 | Flower | 1 | [7]; [9] | n.a. | Not protected | NT | Wild |  |
| 147 | *Malva neglecta* Wallr. | Malvaceae | Herb | 6 | 2 | Flower | 3 | [7]; [8] | Positive | Not protected | LC | Cultivated |  |
| 148 | *Malva sylvestris* L. | Malvaceae | Herb | 10 | 3 | Flower; Leaf; seed | 4 | [1]; [7]; [9]; [12] | Positive | Not protected | LC | Cultivated |  |
| 149 | *Marrubium vulgare* L. | Lamiaceae | Herb | 4 | 2 | Leaf | 1 | [7]; [8]; [9]; [11] | Positive | Not protected | EN | Cultivated |  |
| 150 | *Matricaria chamomilla* L. | Asteraceae | Herb | 13 | 4 | Flower | 3 | [1], [4]; [7]; [8] [9]; [10]; [11]; [12] | positive | Not protected | LC | Cultivated |  |
| 151 | *Melilotus officinalis* (L.) Pall. | Fabaceae | Herb | 6 | 2 | Bud; Flower; Leaf | 2 | [1]; [8]; [9]; [10]; [12] | Positive | Not protected | LC | Wild |  |
| 152 | *Mentha aquatica* L. | Lamiaceae | Herb | 6 | 0 | Leaf | 1 | [4]; [6]; [8] | n.a. | Not protected | LC | Cultivated |  |
| 153 | *Mentha arvensis* L. | Lamiaceae | Herb | 1 | 0 | Leaf | 1 | [4]; [8] | n.a. | Not protected | LC | Wild |  |
| 154 | *Mentha longifolia* (L.) L. | Lamiaceae | Herb | 5 | 2 | Leaf | 4 | [1]; [4]; [8]; [9] | n.a. | Not protected | LC | Cultivated |  |
| 155 | *Mentha pulegium* L | Lamiaceae | Herb | 3 | 1 | Leaf | 2 | [1]; [4]; [7]; [8]; [12] | n.a. | Not protected | CR | Cultivated |  |
| 156 | *Menyanthes trifoliata* L. | Menyanthaceae | Herb | 2 | 2 | Leaf | 1 | [1]; [8] | Positive | Not protected | NT | Wild |  |
| 157 | *Mutellina adonidifolia* (J.Gay) Gutermann | Apiaceae | Herb | 2 | 3 | Root | 4 | [7]; [8]; [9]; [12] | n.a. | Not protected | LC | Wild | x |
| 158 | *Myricaria germanica (L*.*) Desv*. | Tamaricaceae | Shrub | 1 | 1 | Leaf | 1 | [9] | n.a. | protected | EN | Wild |  |
| 159 | *Nasturtium officinale* R.Br. | Brassicaceae | Herb | 7 | 3 | Flower; Leaf; seed | 2 | [1]; [7]; [8]; [9] | Positive | Not protected | NT | Wild |  |
| 160 | *Nepeta cataria* L. | Lamiaceae | Herb | 1 | 0 | Leaf | 1 | [4]; [7] | n.a. | Not protected | NT | Wild |  |
| 161 | *Ononis spinosa* L. | Fabaceae | Shrub | 1 | 0 | Root | 1 | [1]; [11] | Positive | Not protected | LC | Wild |  |
| 162 | *Origanum vulgare* L. | Lamiaceae | Herb | 5 | 2 | Flower; Leaf | 2 | [7]; [8]; [9]; [10] | Negative | Not protected | LC | Cultivated |  |
| 163 | *Ornithogalum umbellatum* L. | Asparagaceae | Herb | 2 | 0 | Flower | 2 | [4] | n.a. | Not protected | LC | Cultivated |  |
| 164 | *Papaver rhoeas* L. | Papaveraceae | Herb | 2 | 3 | Seed | 3 | [4]; [7]; [8] | Negative | Not protected | LC | Cultivated |  |
| 165 | *Parnassia palustris* L. | Parnassiaceae | Herb | 1 | 2 | Flower; Leaf | 2 | [4]; [5]; [8] | n.a. | Not protected | LC | Wild |  |
| 166 | *Petasites hybridus* (L.) "G. Gaertn. B.Mey. & Scherb." | Asteraceae | Herb | 2 | 0 | Leaf; Root | 2 | [9]; [10] | Positive | Not protected | LC | Wild |  |
| 167 | *Peucedanum ostruthium* (L.) W.D.J.Koch | Apiaceae | Herb | 10 | 9 | Flower; Leaf; Root | 4 | [1]; [2]; [6]; [7]; [8] [9]; [10]; [11]; [12] | n.a. | Not protected | LC | Wild |  |
| 168 | *Picea abies* (L.) H.Karst. | Pinaceae | Tree | 6 | 3 | Bark; Bud; Leaf; Resin | 4 | [1]; [6]; [7]; [9] | Positive | Not protected | LC | Wild |  |
| 169 | *Pimpinella major* (L.) Huds. | Apiaceae | Herb | 6 | 2 | Leaf; Root | 3 | [1]; [7]; [8]; [11] | Positive | Not protected | LC | Wild |  |
| 170 | *Pimpinella saxifraga* L. | Apiaceae | Herb | 8 | 8 | Leaf; Root | 3 | [7]; [8]; [11] | Positive | Not protected | LC | Wild | x |
| 171 | *Pinguicula alpina* L. | Lentibulariaceae | Herb | 1 | 3 | Leaf; Root | 3 | [7]; [8] | n.a. | Not protected | LC | Wild |  |
| 172 | *Pinus cembra* L. | Pinaceae | Tree | 6 | 3 | Fruit; Leaf; Resin | 4 | [1]; [4]; [7]; [9]; [10]; [12] |  | Not protected | LC | Wild |  |
| 173 | *Pinus mugo* Turra | Pinaceae | Tree | 7 | 3 | Leaf; Resin | 3 | [1]; [4]; [7]; [9] | n.a. | Not protected | LC | Wild |  |
| 174 | *Pinus sylvestris* L. | Pinaceae | Tree | 3 | 1 | Fruit; Leaf; Resin | 4 | [1]; [6]; [7]; [9] | Positive | Not protected | LC | Wild |  |
| 175 | *Plantago lanceolata* L. | Plantaginaceae | Herb | 14 | 2 | Leaf; Root | 3 | [1]; [5]; [7]; [8]; [9] | Positive | Not protected | LC | Cultivated |  |
| 176 | *Plantago major* L. | Plantaginaceae | Herb | 9 | 2 | Leaf; Root; Seed | 3 | [1]; [5]; [7]; [9] | n.a. | Not protected | LC | Cultivated |  |
| 177 | *Plantago media* L. | Plantaginaceae | Herb | 2 | 0 | Leaf; Root | 1 | [1]; [7]; [9]; [10] | n.a. | Not protected | LC | Wild |  |
| 178 | *Polygala chamaebuxus* L. | Polygalaceae | Herb | 1 | 4 | Flower; Seed | 2 | [7] | n.a. | Not protected | LC | Wild | x |
| 179 | *Polygonatum odoratum* (Mill.) Druce | Ruscaceae | Herb | 1 | 0 | Root | 1 | [10] | n.a. | Not protected | LC | Wild |  |
| 180 | *Polygonum aviculare* L. | Polygonaceae | Herb | 3 | 0 | Leaf | 1 | [7]; [9]; [11] | n.a. | Not protected | LC | Wild |  |
| 181 | *Polypodium vulgare* L. | Polypodiaceae | Fern | 3 | 3 | Root | 2 | [1]; [7]; [8] | n.a. | Not protected | LC | Wild |  |
| 182 | *Populus tremula* L. | Salicaceae | Tree | 2 | 1 | Bark; Bud; Leaf | 2 | [1]; [9] | Negative | Not protected | LC | Wild |  |
| 183 | *Potentilla anserina* L. | Rosaceae | Herb | 10 | 4 | Flower; Leaf | 3 | [4]; [8]; [10]; [11]; [12] | Positive | Not protected | LC | Cultivated |  |
| 184 | *Potentilla aurea* L., | Rosaceae | Herb | 1 | 0 | Flower; Leaf; Root | 1 | [2]; [8], [12] | n.a. | Not protected | LC | Wild |  |
| 185 | *Potentilla erecta* (L.) Raeusch. | Rosaceae | Herb | 7 | 2 | Root | 2 | [8]; [9]; [12] | Positive | Not protected | LC | Wild |  |
| 186 | *Potentilla grandiflora* L. | Rosaceae | Herb | 1 | 0 | Flower; Leaf; Root | 1 | [2] | n.a. | Not protected | LC | Wild |  |
| 187 | *Potentilla reptans* L. | Rosaceae | Herb | 1 | 0 | Leaf; Root | 1 | [2] | n.a. | Not protected | LC | Wild |  |
| 188 | *Primula auricula* L. | Primulaceae | Herb | 1 | 3 | Flower; Leaf | 1 | [7]; [9] | n.a. | Protected | LC | Wild | x |
| 189 | *Primula elatior* (L.) Hill | Primulaceae | Herb | 6 | 10 | Flower; Leaf | 2 | [4]; [6]; [7]; [9]; [10] | Positive | Protected | LC | Wild |  |
| 190 | *Primula farinosa* L. | Primulaceae | Herb | 1 | 3 | Flower; Leaf | 1 | [10] | n.a. | Not protected | LC | Wild |  |
| 191 | *Primula glutinosa* Wulfen | Primulaceae | Herb | 5 | 3 | Flower; Leaf; Root | 1 | [4]; [6]; [7]; [8] | n.a. | Protected | LC | Wild |  |
| 192 | *Primula matthioli* (L.) K.Richt. | Primulaceae | Herb | 1 | 4 | Flower; Leaf | 1 | [4]; [9]; [10] | n.a. | Protected | VU | Wild | x |
| 193 | *Primula veris* L. | Primulaceae | Herb | 9 | 10 | Flower; Leaf; Root | 2 | [4]; [6]; [7]; [9]; [10] | positive | Protected | LC | Wild |  |
| 194 | *Primula vulgaris* Huds. | Primulaceae | Herb | 2 | 2 | Leaf; Root | 1 | [7]; [9] | n.a. | Protected | LC | Wild |  |
| 195 | *Prunus avium* (L.) L. | Rosaceae | Tree | 3 | 1 | Fruit | 2 | [7]; [8] | n.a. | Not protected | LC | Cultivated |  |
| 196 | *Prunus spinosa* L. | Rosaceae | Tree | 5 | 2 | Flower; Fruit | 2 | [1]; [6]; [8]; [11] | Negative | Not protected | LC | Wild |  |
| 197 | *Pteridium aquilinum* (L.) Kuhn. | Dennstaedtiaceae | Fern | 1 | 0 | Leaf; Root | 1 | [1]; [8]; [10] | n.a. | Not protected | LC | Wild |  |
| 198 | *Pulmonaria officinalis* L. | Boraginaceae | Herb | 5 | 0 | Flower; Leaf; Root | 1 | [7]; [11] | Negative | Not protected | LC | Cultivated |  |
| 199 | *Pyrus pyraster* (L.) Burgsd. | Rosaceae | Tree | 2 | 1 | Fruit | 4 | [2]; [6] | n.a. | Not protected | LC | Wild |  |
| 200 | *Quercus petraea* (Matt.) Liebl. | Fagaceae | Tree | 4 | 3 | Bark | 5 | [8]; [9] | Positive | Not protected | LC | Wild |  |
| 201 | *Quercus pubescens* Willd. | Fagaceae | Tree | 2 | 3 | Bark | 5 | [8]; [9] | n.a. | Not protected | LC | Wild |  |
| 202 | *Quercus robur* L. | Fabaceae | Tree | 6 | 2 | Bark | 5 | [8]; [9] | Positive | Not protected | VU | Wild |  |
| 203 | *Ranunculus acris* L. | Ranunculaceae | Herb | 1 | 1 | Leaf | 1 | [10] | n.a. | Not protected | LC | Wild |  |
| 204 | *Beckwithia glacialis* (L.) Á.Löve & D.Löve | Ranunculaceae | Herb | 1 | 3 | Flower; Leaf; Root | 1 | [7]; [10] | n.a. | Not protected | LC | Wild | x |
| 205 | *Raphanus raphanistrum* L. | Brassicaceae | Herb | 2 | 3 | Leaf; Root | 2 | [7]; [8]; [11] | n.a. | Not protected | LC | Wild |  |
| 206 | *Rhamnus frangula* L. | Rhamnaceae | Tree | 1 | 1 | Bark | 1 | [8] | n.a. | Not protected | LC | Wild |  |
| 207 | *Sedum roseum* (L.) Scop. | Crassulaceae | Herb | 3 | 2 | Leaf | 2 | [3]; [4]; [7] | n.a. | Not protected | LC | Wild | x |
| 208 | *Rhododendron ferrugineum* L. | Ericaceae | Shrub | 3 | 3 | Flower | 3 | [7]; [8]; [10] | Negative | Not protected | LC | Wild |  |
| 209 | *Ribes petraeum* Wulfen | Grossulariaceae | Shrub | 5 | 2 | Fruit; Leaf | 2 | [3]; [8]; [10] | n.a. | Not protected | NE | Cultivated |  |
| 210 | *Rosa canina* L. | Rosaceae | Shrub | 12 | 4 | Flower; Fruit; Leaf | 3 | [1]; [2]; [7]; [8]; [9]; [10]; [11] | Negative | Not protected | LC | Cultivated |  |
| 211 | *Rosa corymbifera Borkh*. | Rosaceae | Shrub | 1 | 0 | Flower | 2 | [9] | n.a. | Not protected | LC | Wild |  |
| 212 | *Rosa montana* Chaix ex Vill. | Rosaceae | Shrub | 2 | 1 | Flower; Fruit | 3 | [9] | n.a. | Not protected | EN | Wild |  |
| 213 | *Rosa pendulina* L. | Rosaceae | Shrub | 1 | 3 | Flower; Fruit | 3 | [1]; [7]; [12] | n.a. | Not protected | LC | Wild |  |
| 214 | *Rubus bifrons* Vest | Rosaceae | Shrub | 8 | 1 | Fruit; Leaf; Root | 3 | [6]; [7]; [8]; [9]; [12] | Positive | Not protected | LC | Wild |  |
| 215 | *Rubus idaeus* L. | Rosaceae | Shrub | 6 | 2 | Fruit; Leaf; Root | 2 | [6]; [7]; [8]; [9]; [12] | Negative | Not protected | LC | Cultivated |  |
| 216 | *Rumex acetosa* L. | Polygonaceae | Herb | 2 | 0 | Leaf | 2 | [8] | n.a. | Not protected | LC | Wild |  |
| 217 | *Rumex alpinus* L. | Polygonaceae | Herb | 1 | 0 | Root | 2 | [8] | n.a. | Not protected | LC | Wild |  |
| 218 | *Rumex crispus* L. | Polygonaceae | Herb | 1 | 0 | Root | 1 | [8] | n.a. | Not protected | LC | Wild |  |
| 219 | *Rumex scutatus* L. | Polygonaceae | Herb | 1 | 0 | Leaf | 2 | [8] | n.a. | Not protected | LC | Wild |  |
| 220 | *Ruscus aculeatus* L. | Ruscaceae | Shrub | 1 | 0 | Root | 1 | [7]; [8] | Positive | Partially protected | LC | Wild |  |
| 221 | *Salix alba* L. | Salicaceae | Tree | 5 | 3 | Bark | 4 | [7]; [9]; [10]; [11] | Positive | Not protected | LC | Wild |  |
| 222 | *Salix caprea* L. | Salicaceae | Tree | 4 | 3 | Bark | 4 | [4]; [7]; [9]; [10] | Positive | Not protected | LC | Cultivated |  |
| 223 | *Salix pentandra* L. | Salicaceae | Palm | 1 | 0 | Bark | 1 | [4]; [10] | n.a. | Not protected | NT | Wild |  |
| 224 | *Salix purpurea* L. | Salicaceae | Tree | 3 | 0 | Bark | 2 | [4]; [6]; [9]; [10] | Positive | Not protected | LC | Wild |  |
| 225 | *Salix serpyllifolia* Scop. | Salicaceae | Shrub | 1 | 1 | Seed | 1 | [9]; [10] | n.a. | Not protected | LC | Wild |  |
| 226 | *Salvia glutinosa* L. | Lamiaceae | Herb | 3 | 2 | Flower; Leaf | 2 | [8]; [9] | n.a. | Not protected | LC | Wild |  |
| 227 | *Sambucus ebulus* L. | Adoxaceae | Shrub | 2 | 3 | Root | 2 | [8]; [11] | n.a. | Not protected | LC | Wild |  |
| 228 | *Sambucus nigra* L. | Adoxaceae | Tree | 13 | 3 | Bark; Flower; Fruit; Leaf; Root | 5 | [1]; [2]; [6]; [7]; [8]; [9]; [10]; [11] | Positive | Not protected | LC | Cultivated |  |
| 229 | *Sambucus racemosa* L. | Adoxaceae | Shrub | 1 | 3 | Flower | 2 | [6]; [7]; [8]; [10] | n.a. | Not protected | LC | Wild |  |
| 230 | *Sanguisorba minor* Scop. | Rosaceae | Herb | 1 | 0 | Flower; Leaf | 2 | [8] | n.a. | Not protected | LC | Wild |  |
| 231 | *Sanicula europaea* L. | Apiaceae | Herb | 3 | 4 | Leaf; Root | 1 | [9]; [10] | Positive | Not protected | LC | Wild |  |
| 232 | *Saponaria officinalis* L. | Caryophyllaceae | Herb | 2 | 1 | Root | 2 | [7] | Positive | Not protected | LC | Cultivated |  |
| 233 | *Scrophularia nodosa* L. | Scrophulariaceae | Herb | 1 | 0 | Leaf, Root | 1 | [1]; [7]; [9] | n.a. | Not protected | LC | Wild |  |
| 234 | *Sedum atratum* L. | Crassulaceae | Herb | 1 | 0 | Leaf | 1 | [4]; [9] | n.a. | Not protected | LC | Wild |  |
| 235 | *Sedum telephium* L. | Crassulaceae | Herb | 1 | 0 | Leaf | 1 | [4]; [9] | n.a. | Not protected | LC | Cultivated |  |
| 236 | *Sempervivum montanum* L. | Crassulaceae | Herb | 1 | 3 | Flower; Leaf | 2 | [1]; [9]; [10] | n.a. | Not protected | LC | Wild |  |
| 237 | *Sempervivum tectorum* L. | Crassulaceae | Herb | 8 | 4 | Leaf | 3 | [1]; [9]; [10]; [12] | n.a. | Not protected | LC | Cultivated |  |
| 238 | *Silene acaulis* (L.) Jacq. | Caryophyllaceae | Herb | 1 | 3 | Flower; Leaf; Root | 3 | [3]; [4] | n.a. | Not protected | LC | Wild |  |
| 239 | *Silene vulgaris* (Moench) Garcke | Caryophyllaceae | Herb | 1 | 1 | Flower; Leaf | 2 | [2]; [8] | n.a. | Not protected | LC | Wild | x |
| 240 | *Solanum dulcamara* L. | Solanaceae | Herb | 1 | 3 | Leaf | 1 | [7]; [9]; [10] | positive | Not protected | LC | Wild |  |
| 241 | *Soldanella alpina* L. | Primulaceae | Herb | 1 | 3 | Flower; Leaf; Root | 1 | [4] | n.a. | Not protected | LC | Wild |  |
| 242 | *Solidago virgaurea* L. | Asteraceae | Herb | 9 | 3 | Flower; Leaf | 2 | [1]; [4]; [7], [8]; [9]; [11] | positive | Not protected | LC | Wild |  |
| 243 | *Sorbus aucuparia* L. | Rosaceae | Tree | 7 | 3 | Flower; Fruit | 3 | [2]; [6]; [7], [8]; [11] | negative | Not protected | LC | Wild |  |
| 244 | *Stachys sylvatica* L. | Lamiaceae | Herb | 1 | 3 | Flower; Leaf | 2 | [9]; [10] | n.a. | Not protected | LC | Wild |  |
| 245 | *Stellaria media* (L.) Vill. | Caryophyllaceae | Herb | 5 | 4 | Leaf | 2 | [8]; [9]; [10]; [11 | n.a. | Not protected | LC | Wild |  |
| 246 | *Symphytum officinale* L. | Boraginaceae | Herb | 9 | 3 | Leaf; Root | 3 | [1]; [7]; [9]; [10] | Positive | Not protected | LC | Cultivated |  |
| 247 | *Tanacetum vulgare* L. | Asteraceae | Herb | 1 | 0 | Leaf | 2 | [1]; [4] | Negative | Not protected | LC | Wild |  |
| 248 | *Taraxacum campylodes* G.E.Haglund | Asteraceae | Herb | 13 | 7 | Flower; Leaf; Root | 2 | [2]; [6]; [7], [8]; [9]; [10]; [11] | Positive | Not protected | LC | Wild |  |
| 249 | *Thlaspi rotundifolium* (L.) Gaudin | Brassicaceae | Herb | 1 | 0 | Flower; Leaf | 2 | [11] | n.a. | Not protected | LC | Wild |  |
| 250 | *Thymus pulegioides* L. | Lamiaceae | Herb | 11 | 4 | Flower; Leaf | 5 | [1]; [4]; [6]; [7], [8]; [9]; [10]; [12] | n.a. | Not protected | LC | Cultivated |  |
| 251 | *Tilia cordata* Mill. | Tiliaceae | Tree | 11 | 4 | Flower; Leaf | 2 | [4]; [7], [9]; | Positive | Not protected | LC | Wild |  |
| 252 | *Tilia platyphyllos* Scop. | Tiliaceae | Tree | 10 | 2 | Flower; Leaf | 3 | [4]; [7], [9] | Positive | Not protected | LC | Wild |  |
| 253 | *Tragopogon dubius* Scop. | Asteraceae | Herb | 1 | 1 | Resin | 1 | [9] | n.a. | Not protected | LC | Wild |  |
| 254 | *Trifolium pratense* L. | Fabaceae | Herb | 3 | 4 | Flower; Leaf | 3 | [1], [8]; [9]; [12] | n.a. | Not protected | LC | Wild |  |
| 255 | *Trifolium repens* L. | Fabaceae | Herb | 2 | 1 | Flower; Leaf | 3 | [1], [9]; [11]; [12] | n.a. | Not protected | LC | Wild |  |
| 256 | *Tussilago farfara* L. | Asteraceae | Herb | 9 | 3 | Flower; Leaf | 3 | [1], [7]; [9]; [10] | Positive | Not protected | LC | Wild |  |
| 257 | *Urtica dioica* L. | Urticaceae | Herb | 15 | 5 | Leaf; Root; Seed | 5 | [6]; [7], [8]; [9]; [10]; [11] | Positive | Not protected | LC | Cultivated |  |
| 258 | *Urtica urens* L. | Urticaceae | Herb | 8 | 1 | Leaf; Root; Seed | 5 | [6]; [8]; [9]; [10]; [11] | Positive | Not protected | LC | Wild |  |
| 259 | *Usnea barbata* (L.) F.H. Wigg. | Parmeliaceae | Lichen | 1 | 2 | Leaf | 1 | [7]; [9] | Positive | Not protected | NE | Wild |  |
| 260 | *Usnea dasopoga* (Ach.) Nyl. | Parmeliaceae | Lichen | 2 | 1 | leaf | 3 | [1]; [4]; [7] | n.a. | Not protected | NE | Wild |  |
| 261 | *Vaccinium myrtillus* L. | Ericaceae | Shrub | 7 | 4 | Fruit; Leaf; Root | 3 | [1]; [2]; [5]; [6]; [8] | Positive | Not protected | LC | Wild |  |
| 262 | *Vaccinium vitis-idaea* L. | Ericaceae | Shrub | 6 | 3 | Fruit; Leaf | 3 | [1]; [2]; [4]; [6]; [8]; [10]; [11] | n.a. | Not protected | LC | Wild |  |
| 263 | *Valeriana montana* L. | Valerianaceae | Herb | 2 | 1 | Flower; Root | 2 | [4]; [8]; [10]; [11] | n.a. | Not protected | LC | Wild |  |
| 264 | *Valeriana officinalis* L. | Valerianaceae | Herb | 13 | 10 | Leaf; Root | 5 | [3]; [4]; [6]; [8]; [7] | Positive | Not protected | LC | Cultivated |  |
| 265 | *Veratrum album* L. | Melanthiaceae | Herb | 3 | 3 | Root | 2 | [1] | n.a. | Not protected | LC | Wild |  |
| 266 | *Verbascum densiflorum* Bertol. | Scrophulariaceae | Herb | 11 | 5 | Flower; Root | 3 | [7]; [9] | Positive | Not protected | LC | Cultivated |  |
| 267 | *Verbascum phlomoides* L. | Scrophulariaceae | Herb | 7 | 3 | Flower; Root | 1 | [7] | Positive | Not protected | LC | Wild |  |
| 268 | *Verbascum thapsus* L. | Scrophulariaceae | Herb | 7 | 2 | Flower; Root | 1 | [7]; [9] | Positive | Not protected | LC | Wild |  |
| 269 | *Verbena officinalis* L. | Verbenaceae | Herb | 5 | 3 | Flower; Leaf | 2 | [1]; [4]; [7]; [9]; [12] | Negative | Not protected | LC | Cultivated |  |
| 270 | *Veronica alpina* L. | Plantaginaceae | Herb | 2 | 1 | Flower; Leaf | 1 | [4]; [6]; [7]; [8]; [10] | n.a. | Not protected | LC | Wild |  |
| 271 | *Veronica chamaedrys* L. | Plantaginaceae | Herb | 2 | 9 | Leaf | 3 | [4]; [7]; [8]; [9] | n.a. | Not protected | LC | Wild |  |
| 272 | *Veronica officinalis* L. | Plantaginaceae | Herb | 10 | 3 | Leaf | 2 | [4]; [6]; [7]; [8] | Negative | Not protected | LC | Cultivated |  |
| 273 | *Viola biflora* L. | Violaceae | Herb | 1 | 3 | Flower; Leaf | 3 | [1]; [7] | n.a. | Not protected | LC | Wild | x |
| 274 | *Viola odorata* L. | Violaceae | Herb | 5 | 1 | Leaf; Root | 3 | [2]; [4]; [7]; [9] | Negative | Not protected | LC | Cultivated |  |
| 275 | *Viola tricolor* L. | Violaceae | Herb | 5 | 4 | Flower; Leaf | 1 | [8]; [9]; [10] | Positive | Not protected | LC | Cultivated |  |
| 276 | *Viscum album* L. | Santalaceae | Shrub | 7 | 4 | Leaf | 3 | [6]; [11]; [12] | Positive | Not protected | LC | Wild |  |

^1^Medicinal use spectrum of traditional medicinal plants in South Tyrol, classified into 12 human disorders categories based on the International Classification of Primary Care (ICPC-2) [45]: [1] General and Unspecified; [2] Digestive; [3] Eye; [4] Cardiovascular; [5] Musculoskeletal; [6] Neurological; [7] Psychological; [8] Respiratory; [9] Skin; [10] Endocrine/ Metabolic and Nutritional; [11] Urology; [12] Pregnancy, Childbirth, Family Planning
